# Intramolecular Alkyne Aromatization: Unexpected Synthesis of Expanded [9]Helicene and π‐Extended Double [4]Helicene, and Their Molecular Geometry Effect on Transistor Memory

**DOI:** 10.1002/smsc.202300040

**Published:** 2023-06-29

**Authors:** Yang Yu, Le Wang, Chang Wang, Fei Liu, Haifeng Ling, Junzhi Liu

**Affiliations:** ^1^ Department of Chemistry and State Key Laboratory of Synthetic Chemistry The University of Hong Kong Hong Kong 999077 China; ^2^ State Key Laboratory of Organic Electronics and Information Displays & Institute of Advanced Materials (IAM) Nanjing University of Posts & Telecommunications Nanjing 210023 China

**Keywords:** alkyne aromatization, helicenes, molecular geometry, molecular packing, polycyclic aromatic hydrocarbons, transistor memories

## Abstract

Intramolecular alkyne aromatization is a powerful tool that enables the synthesis of nonplanar polycyclic aromatic hydrocarbons. Herein, an unexpected intramolecular alkyne aromatization via a trifluoroacetic acid‐promoted cyclization is described, in which two structural isomers, expanded [9]helicene (**1**) and π‐extended double [4]helicene (**2**) are obtained. A possible rearrangement mechanism is proposed to account for the formation of **1**. The geometric and optoelectronic properties of these two nonplanar molecular nanocarbons are comprehensively investigated by single‐crystal X‐ray, UV–vis absorption, photoluminescence spectra, and cyclic voltammetry analysis. These two structural isomers exhibit wide energy gaps with similar energy levels, which further apply them as molecular floating gate in organic field‐effect transistor nonvolatile memory (OFET‐NVM) devices. The nonplanar geometry of **1** and **2** shows a remarkable effect on charge‐trapping behaviors in OFET‐NVMs; the **1**‐based device displays a more than threefold wider memory window (MW, 44.5 V) than that of the **2**‐based device (14.2 V), and a large charge‐trapping density of 1.08 × 10^13^ cm^−2^. The distinct different charge‐trapping behavior is likely attributed to the different molecular geometries, resulting in different molecular‐packing modes. It is revealed in this study that controlling the geometry of molecular nanocarbons is a new strategy for application in organic electronics.

## Graphical abstract

An unexpected intramolecular alkyne aromatization enables the synthesis of two isomers, expanded [9]helicene and π‐extended double [4]helicene. Single‐crystal X‐ray analysis reveals their different helical structures and packing modes. Although they have nearly identical highest occupied molecular orbital/lowest unoccupied molecular orbital levels, the molecular geometry significantly affects charge‐trapping behavior in organic field‐effect transistor memory.

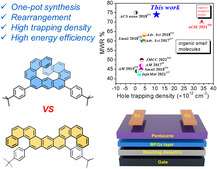

## 1 Introduction

Polycyclic aromatic hydrocarbons (PAHs) have emerged as appealing alternatives to inorganic semiconductors against the background of the intelligent era due to their structural tailorability, processability, and diversity.^[^ ^1^, ^2^, ^3^, ^4^ ^]^ The electronic structures of PAHs can be finely tailored by the molecular sizes, edge structures, and molecular geometries, allowing for tuning their solubility, optoelectronic properties, and self‐assembly behavior.^[^ ^5^, ^6^ ^]^ Among them, nonplanar PAHs exhibit excellent optical, electronic, and magnetic properties due to their rigid twisted π‐systems and unique self‐assembly behavior, endowing them as promising candidates in the fields of optoelectronics, nonlinear optics, and spintronics.^[^ ^7^, ^8^, ^9^, ^10^, ^11^ ^]^ Helicenes are a kind of aromatic *ortho*‐condensed polycyclic compounds with angularly annulated structures. Incorporating more benzene rings into carbohelicenes results in the extension of the π‐system to form expanded helicenes or π‐extended helicenes, which can lead to unique geometries and optoelectronic properties.^[^ ^12^, ^13^, ^14^, ^15^, ^16^, ^17^ ^]^ The helicenes with different topological geometries have shown potential application in optoelectronic devices, such as circularly polarized photodetectors,^[^ ^18^, ^19^, ^20^ ^]^ light‐emitting diodes,^[^ ^21^ ^]^ spin filters,^[^ ^22^ ^]^ biological sensors,^[^ ^23^ ^]^ and memories.^[^ ^24^ ^]^

The organic field‐effect transistor (OFET) is an essential platform for organic electronics, especially, for the applications in data storage, sensor arrays, light‐emitting devices, and flexible logic circuits.^[^ ^25^, ^26^, ^27^, ^28^, ^29^, ^30^ ^]^ Incorporating one additional floating gate in the structure of OFET to form the OFET nonvolatile memory (OFET‐NVM) has shown promise in flexible electronics due to its simple structure and high memory performance.^[^ ^31^, ^32^, ^33^ ^]^ In recent years, molecular floating gates (MFGs) have been rapidly developed due to their superiorities of precise electronic structure, consistency of size and morphology.^[^ ^34^ ^]^ Different kinds of MFGs have been explored, including acene,^[^ ^35^ ^]^ fullerene^[^ ^36^, ^37^ ^]^ and imide derivatives,^[^ ^38^, ^39^ ^]^ donor–accepter small molecules,^[^ ^40^ ^]^ organic metal complexes,^[^ ^41^ ^]^ and nanographenes,^[^ ^42^ ^]^ to meet the specific requirements of OFET‐NVMs by tailoring their molecular structures. However, manipulating the charge‐trapping behavior of MFGs is challenging, which requires further understanding of the structure–property relationship and the microstructure of charge‐trapping molecules. Although studies on the structure–property relationship in MFGs are limited, some useful design strategies have been proposed, such as the influence of energy levels,^[^ ^43^ ^]^ dipole moments,^[^ ^44^ ^]^ molecular symmetry,^[^ ^45^ ^]^ and steric hindrance,^[^ ^40^ ^]^ etc. However, the role of molecular geometry on transistor memory is often overlooked. In this regard, nanographenes bearing helical structures are expected to be potential materials for MFGs in OFET‐NVMs to investigate the molecular geometry effect, which is scarcely studied in transistor memory.

Exploring cyclization methods to prepare molecular structures with nonplanar geometries and applying them in organic devices is of great importance for synthetic chemistry and organic materials.^[^ ^46^, ^47^, ^48^ ^]^ The annulation methods for helical structures, such as photocyclization,^[^ ^49^ ^]^ olefin metathesis cyclization,^[^ ^50^ ^]^ Scholl reaction,^[^ ^13^ ^]^ and alkyne benzannulation,^[^ ^14^, ^51^ ^]^ have been widely explored in last decade. In this study, one expanded [9]helicene (**1**) and one π‐extended double [4]helicene (**2**), which are structural isomers, were obtained from the same precursor (**4**) through an unexpected intramolecular alkyne aromatization. Single‐crystal X‐ray analysis unambiguously revealed the helical structures of compounds **1** and **2**, as well as their packing modes. UV–vis absorption and cyclic voltammetry (CV) analysis, in combination with the density‐functional theory (DFT) calculations, disclosed the wide energy gaps for **1** and **2**. Moreover, these two structural isomers were further explored as MFGs in OFET‐NVM devices. Charge‐trapping layers (CTLs) with a thickness of 20 nm were successfully fabricated using a solution spin‐coating process, which is large enough to inherit the structural motifs observed in the bulk crystal structure. Although the two molecules have nearly identical highest occupied molecular orbital (HOMO) and lowest unoccupied molecular orbital (LUMO) energy levels, we found that the molecular geometry has a significant impact on charge‐trapping behavior in OFET‐NVMs. Compound **2**–based device exhibited a memory window (MW) of about 14.2 V. In contrast, **1**‐based device showed a more than threefold wider MW of 44.5 V than that of **2** and a very large charge‐trapping density (1.08 × 10^13 ^cm^−2^ for **1** and 3.4 × 10^12 ^cm^−2^ for **2**), this is likely owing to the different packing modes at the solid state induced by their molecular geometry. In addition, a large ON/OFF ratio (≈10^5^), long‐term charge retention (>10^4^ s) and reliable endurance (800 cycles) are obtained for **1**‐based device. Our studies demonstrated that the molecular geometry effect on charge trapping highlights the importance of understanding the relationship between molecular structure and electronic properties. The results reveal that controlling the geometry of molecular nanocarbons could be an alternative strategy for applications in organic electronics.

## 2 Results and Discussion

**Scheme 1:**
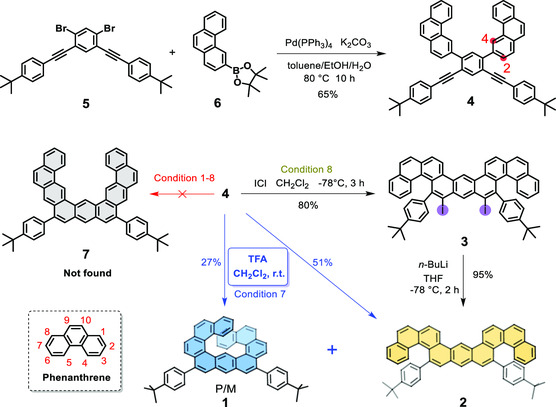
Schematic illustration of the synthesis of molecules **1** and **2**.

**Figure 1:**
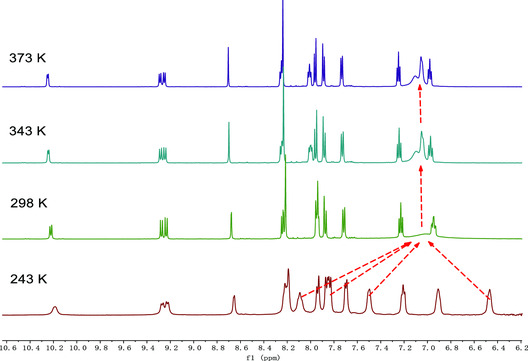
Variable‐temperature ^1^H NMR spectra of **2** (600 MHz, C_2_D_2_Cl_4_) in aromatic region. The temperature ranges from 243 to 373 K.

The synthesis of molecules **1** and **2** is described in **Scheme** **1**. The starting materials of 4,4'‐((4,6‐dibromo‐1,3‐phenylene)bis(ethyne‐2,1‐diyl))bis(*tert*‐butylbenzene) (**5**) and 4,4,5,5‐tetramethyl‐2‐(phenanthren‐3‐yl)‐1,3,2‐dioxaborolane (**6**) were prepared according to the synthetic routes described in Scheme S1, Supporting Information. Compound **4** was obtained from **5** and **6** via a twofold Suzuki cross‐coupling reaction with a yield of 65%. Initially, we intuitively considered that the proposed U‐shaped compound **7** could be formed during the twofold alkyne benzannulation of precursor **4**. To our surprise, compound **7** has never been found under these conditions (Table S1, Supporting Information). It might be attributed to the regioselective reaction of the phenanthryl units,^[^ ^13^, ^52^, ^53^ ^]^ which could be rationalized by the higher reactivity of the 4‐positions with a more significant electron density over the 2‐positions of phenanthrene (Scheme 1, Table S1, Supporting Information, entry 8). Then, iodine monochloride (ICl)‐induced cyclization was performed. The iodine‐substituted intermediate **3** was obtained in good yield (80%) by treatment of **4** with ICl under −78 °C. Next, the iodo‐substituents were removed using *n*‐BuLi to afford **2** in a high yield of 95%. The successful formation of **3** could be attributed to the rotation of the phenanthryl units of **4** in the selective ICl‐induced cyclization under a low temperature without forming significant amount of other cyclized products. The structure of **2** was verified by variable‐temperature nuclear magnetic resonance (NMR) analysis (**Figure** **1**, S1, and S2, Supporting Information). In other ways, Brønsted acid was also investigated for activating the alkyne aromatization of **4**.^[^ ^54^, ^55^ ^]^ Upon treatment of **4** with triflic acid (TfOH) at room temperature, compound **2** (yield 21%) and another blue light product **1** was obtained (Table S1, Supporting Information, entry 6). To our surprise, characterizations of the blue light product **1** by high‐resolution mass spectrometry (HRMS), 1D‐ and 2D‐NMR, and single‐crystal X‐ray analysis revealed its unexpected expanded [9]helicene structure through an rearrangement (Scheme 1). Finally, the reaction was optimized by treatment of **4** with excess trifluoroacetic acid (TFA), the cycloisomerization reaction smoothly occurred. The yields of compounds **1** and **2** were 27% and 51%, respectively (Table S1, Supporting Information, entry 7). The proposed possible mechanism for the formation of **1** is described in Scheme S2 (see Supporting Information).

The low field shift of singlet peaks of the center benzene ring in ^1^H‐NMR and the disappearance of the acetylenic resonances at ≈94 and 88 ppm of precursor **4** in the ^13^C‐NMR spectrum essentially indicate the successful twofold alkyne aromatization of **1** and **2** (Figure S1 and S2, Supporting Information). Compound **1** shows a clear ^1^H‐NMR spectrum, which reveals its highly symmetric structure (Figure S13, Supporting Information). And with the help of ^1^H/^1^H correlation spectroscopy (COSY) and nuclear Overhauser effect spectroscopy (NOESY) analysis, each proton of **1** could be assigned (Figure S14 and S15, Supporting Information). Compound **2** shows complicated ^1^H‐NMR spectrum, probably because it possesses interconvertible dynamic structures. For the helical structure of **2**, the site exchange related to the *tert*‐butyl benzene rings and [4]helicene segments should result from the helical inversions, namely, the exchange between the enantiomeric helical structures or the rotation of *tert*‐butyl benzene rings. The dynamic behavior of the double‐helical structures was investigated by temperature‐dependent NMR measurements (Figure 1). For molecule **2**, owing to the rotation of the *tert*‐butyl benzene rings restricted by the [4]helicene segments, the peaks of proton signal are broadened under room temperature, however, collapsed together under high temperature (343 K). Under low temperature (243 K), the protons of the *tert*‐butyl benzene rings are changed into four peaks. This result suggests that the *tert*‐butyl benzene rings can interconvert rapidly on the NMR timescale.^[^ ^56^ ^]^ In addition, with the help of ^1^H/^1^H COSY spectrum analysis, each proton of **2** could be assigned (Figure S18, Supporting Information). The high‐resolution electrospray ionization (ESI) mass spectrum of **2** showed two intense peaks with their experimental *m*/*z* value and isotopic distribution precisely matching that predicted for the **2** and its dimer, which indicates a strong intermolecular dimerization (Figure S24 and S25, Supporting Information).

**Figure 2:**
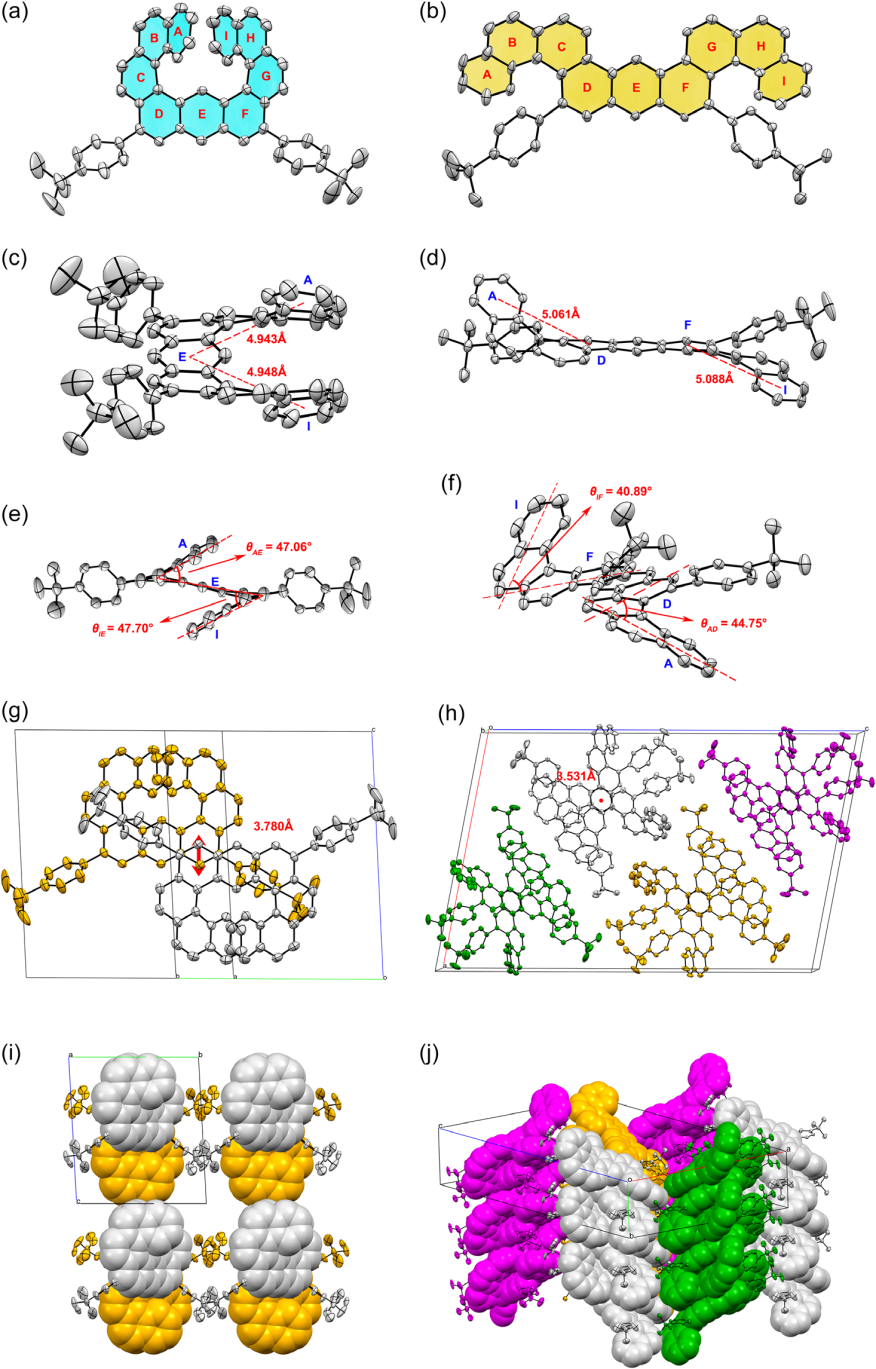
a,b) X‐ray crystallographic molecular structures of **1** (a) and **2** (b), in which hydrogen atoms are omitted for clarity. c,d) The distance between the centroids of the terminal rings of helicenes in **1** (c) and **1** (d). e,f) The splay angles between the two planes of **1** (e) and **2** (f). g,h) The intermolecular π–π distance of **1** (g) and **2** (h). i,j) The packing patterns of **1** (i) and **2** (j). The asymmetric units in one unit cell are shown in different colors. In (i), white and yellow are opposite molecular helicities. In (j), there are also two helicities times two symmetrically independent molecules, which are shown as 4 different colors (yellow, white, green, and red).

The X‐ray diffraction structures of **1** (CCDC 2 244 909) and **2** (CCDC 2 244 910) were investigated, and the specific data is shown in Table S2, Supporting Information. They crystallize in the *P* $\bar{1}$ for **1** and *P2*_*1*_ */c* for **2** space group, respectively. As shown in **Figure** **2**a,b, molecule **1** shows an expanded [9]helicene structure, and **2** displays a π‐extended double [4]helicene geometry. In compound **1**, there are two [5]helicene segments at both ends of the molecule through sharing the ring E, which gives the molecule an “ox horn”–like structure (Figure 2a). The distance between the centroids of the terminal rings A and E and I and E are measured to be 4.943 and 4.948 Å (Figure 2c), respectively, which are highly similar to [5]helicene (CCDC 1 966 144, 4.990 Å). Additionally, the splay angles between the two planes of the terminal rings ($\theta_{\text{AE}}$ and $\theta_{\text{IE}}$) are measured to be 47.06° and 47.70°, respectively (Figure 2e). In compound **2**, two [4]helicene segments are formed at both ends of the molecular structure, which gives an “argali horn” like geometry, which shows the same helicity. The distance between the centroids of the terminal rings A and D and F and I are measured to be 5.061 and 5.088 Å (Figure 2d), respectively, which are similar to that in [4]helicene (CCDC 2 107 530, 5.154 Å). However, the splay angles between the two planes of the terminal rings ($\theta_{\text{AD}}=$ 44.75° and $\theta_{\text{FI}}=$ 40.89°, Figure 2f) are obviously bigger than that in [4]helicene (27.26°), which can be attributed to the influence of the steric hindrance of *tert*‐butylbenzene groups.

As shown in Figure 2g,i, there are two enantiomers in a lattice of **1**. They are distributed in a dislocated “layer to layer” arrangement in space, showing the closest intermolecular distance of 3.78 Å, suggesting a weak intermolecular π–π interaction. However, in the lattice of **2**, eight molecules are interspersed in pairs as dimers. Additionally, along the *b*‐axis (Figure 2h), the intermolecular π–π distance is measured to be 3.531 Å, suggesting a strong intermolecular π–π stacking interaction in **2**. Moreover, molecule **2** shows a great tendency to bind forming dimers, which is also reflected in the difference in crystal density of the two materials (*ρ*_1_ = 0.898 g cm^−3^ and *ρ*_2_ = 1.185 g cm^−3^) (Table S2, Supporting Information). This phenomenon is also consistent with the dimer of **2** in HRMS results (Figure S25, Supporting Information). These results indicate that the molecular geometry of **1** shows an approximate single nanomolecular behavior, which can be regarded as isolated MFGs, and beneficial for charge trapping in OFET‐NVMs.

**Figure 3:**
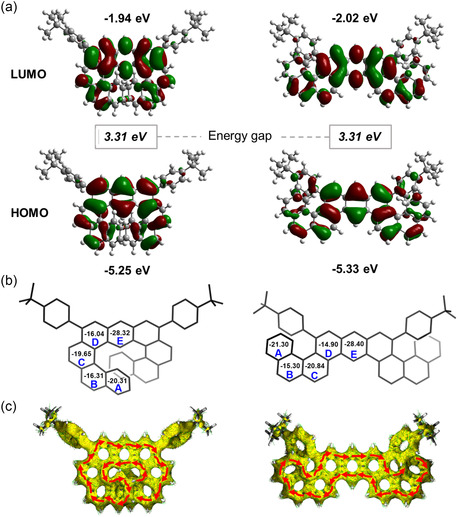
a) Optimized molecular geometries and frontier molecular orbitals of **1** and **2**. b) NICS(1)_zz–avg_ values (calculated at the GIAO‐B3LYP/6‐311 + G(2*d*,*p*) level of theory) and c) calculated ACID plots of **1** and **2**.

As shown in **Figure** **3**a, for both **1** and **2**, the HOMO and LUMO are well distributed on the main backbones. The helical π‐extensions of **1** and **2** show different molecular geometry but with the same overlap of hole and electron in the central anthracene core. The HOMO energy levels of **1** and **2** were calculated to be −5.25 and −5.33 eV, and the LUMO energy levels of **1** and **2** were estimated to be −1.94 and −2.02 eV, respectively. The local aromaticity of the compounds was investigated by the nucleus‐independent chemical shift (NICS) calculated at the gauge invariant atomic orbital‐Becke's three‐parameter hybrid exchange functionals and the Lee–Yang–Parr correlation functional (GIAO‐B3LYP)/6‐311 + g (2*d*,*p*) level. As shown in Figure 3b, both molecules **1** and **2** display global aromaticity along with the same local aromatic trends, indicating that the π‐electrons are delocalized over the molecules. The geometry does not have a distinct effect on the aromaticity in this system. In addition, the results of anisotropy of the induced current density (ACID) calculation show an unbroken clockwise ring current across the whole molecular skeleton, which further supported the global aromaticity of molecules **1** and **2** (Figure 3c). The results show that although compounds 1 and 2 possess different molecular geometry, they still maintain the electronic structure and aromaticity and keep the same energy gap (cal. 3.31 eV), which makes it possible to study the effect of molecular geometry effect independently rather than band engineering in OFET‐NVMs.

**Figure 4:**
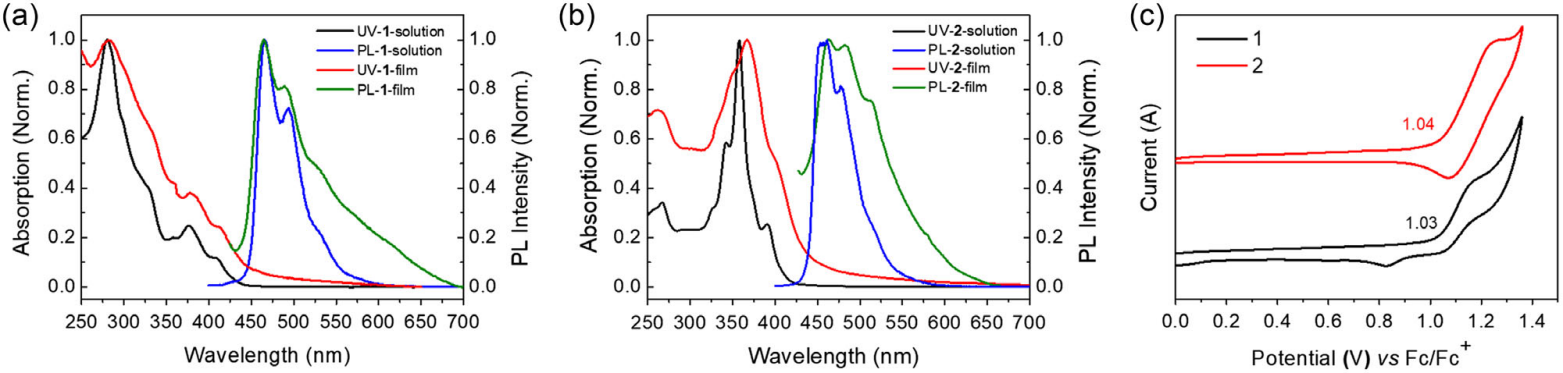
a,b) UV–vis absorption and fluorescence spectra of **1** (a) and **2** (b) in dichloromethane (DCM) (*c* = 1.0 × 10^−5^ m) solution and in thin film, respectively. c) Cyclic voltammograms of **1** and **2** in DCM with 0.1 m [*n*Bu_4_N]PF_6_ as supporting electrolyte and Fc/Fc^+^ as the internal reference at a scan rate of 100 mV s^−1^.

The photophysical properties of **1** and **2** were investigated by UV–vis absorption and photoluminescence (PL) analysis in both solution and films (**Figure** **4a** **,**b), the data were summarized in **Table** **1**. In the solution, for molecule **1**, an intense high‐energy absorption peak at 280 nm with a shoulder at 330 nm and two lower weak absorption peaks at 377 and 409 nm are observed. Molecule **2** shows an absorption peak at 358 nm with two shoulders at 342 and 391 nm. In the visible regions, the main absorption bands arise from the transitions of HOMO to LUMO energy levels. The optical energy gaps (*E*_g_^opt^) of **1** and **2** were calculated to be 2.90 and 3.05 eV, respectively, based on the onset optical absorption (*λ*_onset_^1^ = 427 nm, *λ*_onset_^2^ = 407 nm). The fluorescence spectrum of **1** exhibits a blue‐emission band with a maximum peak at 466 nm and a shoulder peak at 494 nm. Accordingly, the Stokes shift is approximately 57 nm, suggesting a structural change in the excited state relative to the ground state. While molecule **2** shows a broad‐emission band at 457 nm with a shoulder peak at 477 nm, and a Stokes shift of 66 nm. As shown in Figure 4a,b, the absorption and emission bands of **1** and **2** are broadened in the spin‐coated thin film. Interestingly, **1**‐based film shows the same absorption and emission peaks without bathochromic shift. But for **2**‐based film, 10 nm bathochromic shift occurred in both absorption and emission compared to its solution measurements, indicating strong intermolecular interactions in the solid state for **2**. This phenomenon is consistent with the pattern of the crystal packing, and further clarifies that molecule **1** has a much weaker intermolecular interaction in the film state than that of **2**, in which the molecular geometry plays a significant role in regulating intermolecular interaction.

**Table 1:** Photophysical and electrochemical properties of **1** and **2**

Moleculea)	*λ*_abs_, [max nm^−1^]		*λ*_PL_, [max nm^−1^]		*E*_g_^opt^ [eV]	*E*_HOMO_ [eV]	*E*_LUMO_ [eV]
	Solution	Film	Solution	Film			
**1**	280/330/377/409	280/330/377/409	466/494	446	2.90	−5.83	−2.93
**2**	342/358/391	352/368/401	457/477	467/487	3.05	−5.84	−2.79

a) The HOMO energy levels were estimated according to the equation *E*_HOMO_ = −(*E*_ox_^onset^ − *E*_Fc_ + 4.8) eV. The LUMO energy levels were calculated by using the equation of *E*_LUMO_ = *E*_HOMO_ + *E*_g_^opt^.

In the CV measurements, compound **1** shows one quasi‐reversible oxidation, and compound **2** exhibits a reversible oxidation wave (Figure 4c). Accordingly, using the onset oxidation potential (1.03 V for **1** and 1.04 V for **2**), the HOMO energy levels of **1** and **2** were estimated to be −5.83 and −5.84 eV, respectively. The reduction is irreversible, and no reduction waves are found within the electrochemical window. Therefore, the LUMO energy levels (−2.93 eV for **1** and −2.79 eV for **2**) were calculated by using the equation of *E*_LUMO_ *= E*_HOMO_ *+ E*_g_^opt^. As a result, compounds **1** and **2** exhibit almost the same HOMO levels and wide energy gaps, which are favorable for charge trapping in OFET‐NVMs (Table 1).

**Figure 5:**
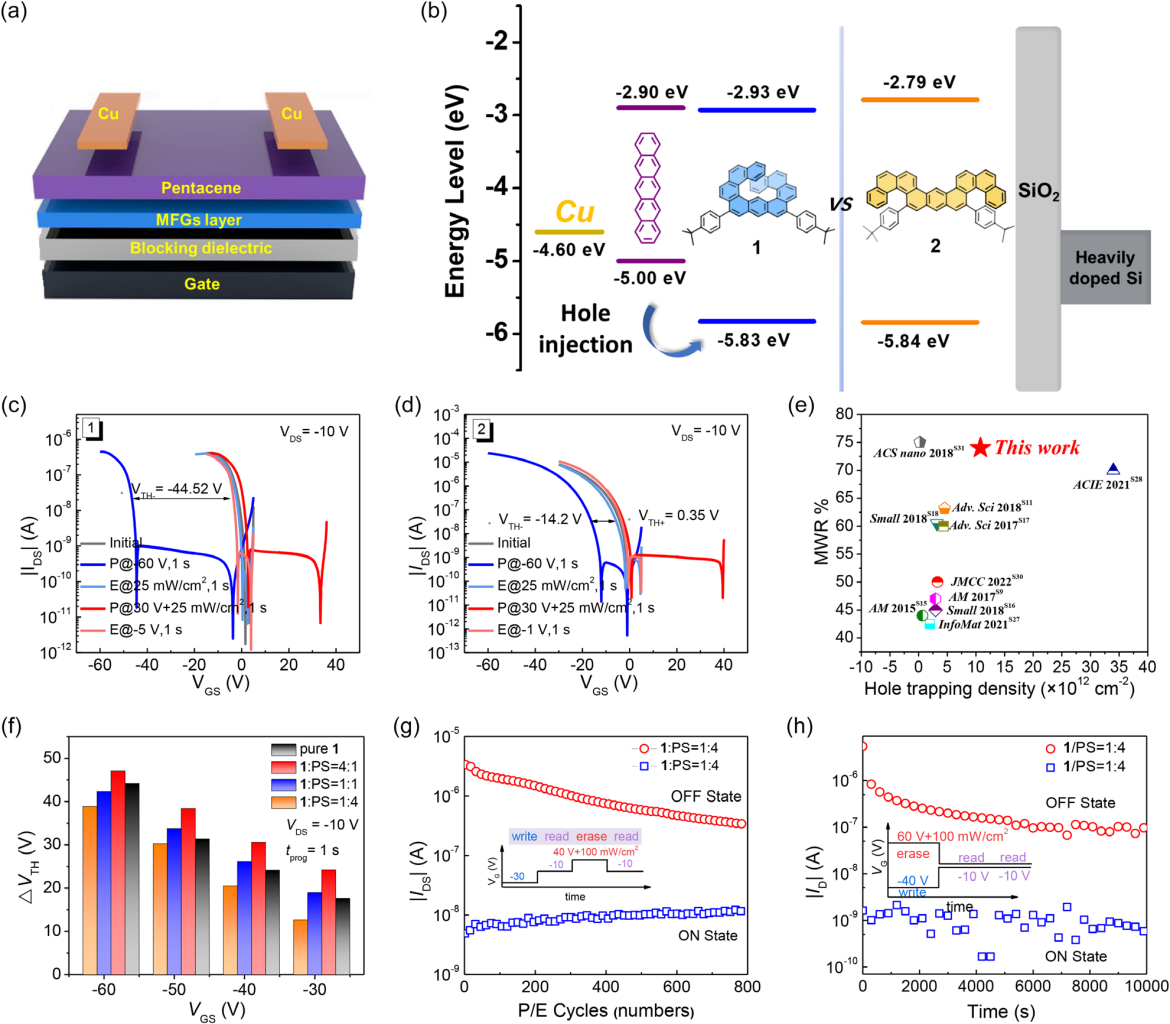
a) Schematic diagram of the pentacene‐based organic field‐effect transistor nonvolatile memory (OFET‐NVM) device with **1** and **2** as molecular floating gates (MFGs). b) Energy levels diagram of Cu electrode, pentacene channel, and MFGs. c,d) Transfer curves of OFET–NVM devices for the programming (P) and erasing (E) processes in both negative and positive gate voltages with devices based on **1** (c) and **2** (d), respectively. e) Comprehensive comparison of memory window ratio (MWR) and hole‐trapping density (Δ*n*) of the pure **1**‐based device with that of the state‐of‐the‐art OFET memory devices reported in recent years. f) MWs (Δ*V*_TH_) as a function of negative *V*_GS_ of pure **1**‐based and different ratios (**1**:PS = 4:1, 1:1, 1:4) blended devices. Δ*V*_TH_ were measured under the operation of various negative *V*_GS_ of −30, −40, −50, and −60 V. g) Endurance characteristics of the **1**:PS = 1:4 based memory device. Reversible switching behavior of the memory device over a series of programming, reading, and erasing processes (inset). h) Retention characteristics of the **1**:PS = 1:4 based device after the writing and erasing operation maintained over 10 000 s.

To gain deeper insight into the molecular geometry effect on memory performance, pentacene‐based OFET‐NVM devices were fabricated (**Figure** **5**a). A spin‐coated film of molecules **1** and **2** (≈20 nm thick) is employed as the MFGs layer, respectively. Films based on molecules **1** and **2** offer hydrophobic interfaces with water contact angles of 100.3° and 95.6°, respectively (Figure S3, Supporting Information), which benefits the growth of pentacene semiconductor films. The transfer characteristics of the memory devices with **1** and **2** exhibit typical p‐type field‐effect behavior with mobility of 0.05 and 0.053 cm^2 ^V^−1 ^s^−1^, respectively (Figure S4, Supporting Information). The corresponding data are summarized in Table S3, Supporting Information. For an OFET‐NVM, the energy barrier between the semiconductor channel and MFGs dominates the efficiency and probability of charge tunneling. As shown in Figure 5b, molecules **1** and **2** have nearly the same hole‐injection barrier. Under the same energy band structure, we investigate the charge‐trapping ability of molecules by measuring the MW (Δ*V*_TH_), which is defined as the difference between the threshold voltage (*V*_TH_) of the programmed (P) and erased (E) states. When a negative gate bias was applied (*V*_GS_ = −60 V, *V*_DS_ = −10 V, 1 s) on the **1**‐based device under dark conditions, the transfer curve significantly shifted in the negative direction, serving as the “programming” process. Furthermore, when light irradiation (power density 25 mW cm^−2^, *V*_GS_ = 0 V) was applied, such device proceeded “erasing” process and recovered to the initial state. As a result, a large MW of 44.5 V could be achieved in the **1**‐based device (Figure 5c). However, for the **2**‐based device, the shift of the transfer curve displayed a small MW of 14.2 V under the same operation (Figure 5d). Interestingly, for these two devices, the detrap or neutralization process cannot realize even at a high positive programming voltage. This erasing process revealed the crucial role of the light illumination in completely releasing the trapped holes, probably via charge–exciton annihilation at the MFG/pentacene heterojunction upon light exposure. The proposed charge storage mechanism is illustrated in Figure S5, Supporting Information. However, both **1**‐ and **2**‐based devices cannot achieve the electron‐trapping mode under positive gate bias, even with the assistance of light irradiation (Figure 5c,d). This is likely owing to the pure carbon aromatic molecular skeleton of **1** and **2**, which lacks electron‐trapping sites, though they have small electron injection barriers. In additional, as shown in Figure S6, Supporting Information, when applying an increasing negative gate bias ranging from −30, −40, −50 to −60 V, the transfer curves of the **1**‐based device realized stepwise negative shifts. It showed multibit MWs of 17.66, 24.13, 31.36, and 44.52 V, which indicates that such a device could perform multibit‐storage function to achieve high storage density in one cell. It is worth mentioning that **1**‐based device shows a hole memory window ratio (MWR, defined as the ratio of Δ*V*_TH_ to programming *V*_GS_) of 74%, and exhibiting a larger hole‐trapping capability and energy efficiency than other reported small molecule‐based MFGs (Figure 5e and Table S4, Supporting Information). The corresponding hole‐trapping density (Δ*n*) of **1**‐based memory is estimated to be 1.08 × 10^13^ cm^−2^ (calculated according to the equation Δ*n* = Δ*V*_TH_ *C*_*i*_/*e*, where *e* is the elementary charge, and *C*_*i*_ is the total capacitance per unit area of the bilayer dielectrics). The results indicate that **1**‐based OFET‐NVM exhibits a good charge‐trapping capability, higher than most of the previously reported state‐of‐the‐art small molecular‐charge‐trapping elements (Figure 5e and Table S4, Supporting Information).

Therefore, the crucial issue of the hole‐trapping capability in these two molecules **1** and **2** strongly depends on their molecular geometry and intermolecular interaction at the solid state. As we discussed in the single crystal and UV–vis–PL spectra, with the unique nonplanar topological configuration, molecule **1** showed weaker intermolecular interaction in the solid state, which can be viewed as isolated MFGs and beneficial for charge trapping in OFET‐NVMs. While for molecule **2**, the stacking columns of the molecule in the solid state may be more conducive to 1D charge transporting rather than charge trapping. Therefore, the differences in stacking patterns and intermolecular interaction induced by the molecular geometry could directly impact on charge‐trapping properties in OFET‐NVMs, which allows us to understand the structure–property relationship of charge‐trapping materials.

**Figure 6:**
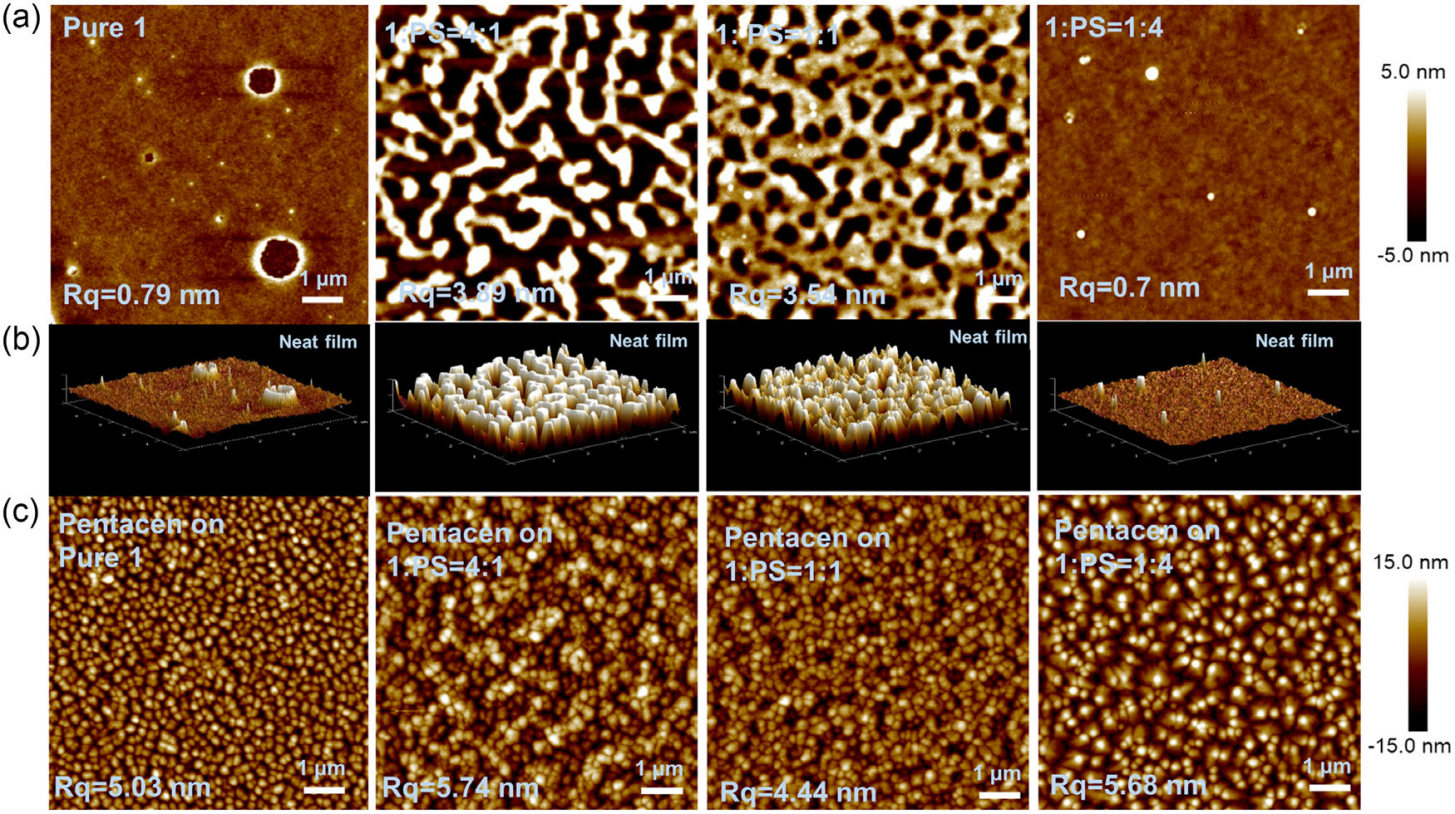
a) 2D atomic force microscopy (2D‐AFM) topographic images of films based on pure **1**, **1**:PS = 4:1, **1**:PS = 1:1, and **1**:PS = 1:4 (from left to right) by solution spin‐coating process on bare 90 nm SiO_2_ substrate. b) The 3D‐AFM images of the previous corresponding thin films. c) The 2D‐AFM topographic images of pentacene on the above different surfaces. Scale bars: 1 μm.

To further clarify the intrinsic molecular‐charge‐trapping property, we blended compound **1** into polystyrene (PS) matrix for fabricating OFET‐NVMs to exclude the morphology effect of the CTLs. In addition, PS could act as a “blocking” layer and improve the charge‐retention property by blocking the charge release,^[^ ^57^, ^58^ ^]^ while not improving memory capacity (Figure S7, Supporting Information). The thin‐film morphologies of the CTLs were investigated by atomic force microscopy (AFM). **Figure** **6**a,b shows the AFM topographic images of the films with pure **1** and different blend ratios with PS (**1**:PS = 4:1, 1:1, 1:4). The 2D and 3D AFM clearly reveal a relatively smooth surface of pure **1** with average root‐mean‐square (RMS) roughness values of 0.79 nm and different degrees of phase separation of blend films (**1**:PS = 4:1 and 1:1) with RMS roughness values of 3.89 and 3.54 nm, respectively. After reducing the blend ratio to 1:4, molecule **1** is well distributed in the PS matrix, and the film presents a uniform and smooth surface with an RMS roughness value of 0.7 nm. AFM images of pentacene on top of the CTLs are shown in Figure 6c. The pentacene deposited on the **1**:PS = 1:4 film exhibits a bigger grain domain with an average size of approximately 0.3 μm than those of other films. The transfer and output curves based on the blend CTLs are shown in Figure S8, Supporting Information. As expected, the drain–source current of the **1**:PS = 1:4 based transistor has been enhanced dramatically, which benefits a larger ON/OFF ratio of 3.38 × 10^5^ and a higher mobility of 0.18 cm^2 ^V^−1 ^s^−1^ than those of other devices. The memory characteristics of the blend CTLs are shown in Figure S9, Supporting Information. Predictably, the blend devices exhibit similar MWs compared with the pure **1**‐based device. Figure 5f shows the MW (Δ*V*_TH_) as a function of applied negative *V*_GS_ of pure **1** and different ratios of blend CTLs. All the devices show a stepwise increased Δ*V*_TH_ with enhanced negative programming bias, corresponding to the Fowler–Nordheim tunneling model. For the **1**:PS = 1:4 based device, the MWs show slightly smaller than pure **1**‐based and high ratio blend devices, ascribed to the reduced amount of the MFGs and total trapping sites in the blend elements. For the pure **1**‐based and blend devices, under the −60 V gate pulse, we measured the corresponding Δ*V*_TH_ with the pulse width (*t*_prog_) changing from 1 ms to 1 s in this process (Figure S10, Supporting Information). The trapping dynamic control for the pure **1**‐based device shows much more dependence on *t*_prog_ (a longer period of pulse > 0.5 s) to get a larger trapping density than blend devices, suggesting that the pure **1** has a larger capacity for charge storage when extending the *t*_prog_ into a larger pulse width, which corresponds to the amount of the MFGs. The larger MWs of blend devices in short pulse range may ascribe to their superior hole mobilities. The results suggested that the memory performance is mainly determined by the intrinsic charge‐trapping capability of the molecule. Furthermore, the endurance and retention characteristics of the memory were investigated to evaluate the reliability and long‐term nonvolatility. The switching stability of the **1**:PS = 1:4 based device was evaluated through write–read–erase–read (WRER) cycles. After applying successive writing and erasing operations, the ON and OFF state currents were steady. As shown in Figure 5g, the device displays reversible WRER behavior with a distinguishable *I*_ON_/*I*_OFF_ ratio after 800 cycles. A degradation in the OFF‐state current of the WRER cycles can be ascribed to a strong hole‐trapping ability of compound **1**, resulting in it not completely returning to the original state after multiple cycles and showing decay. Figure 5h shows the retention time of the programmed and erased states. For the **1**:PS = 1:4 based device, both the ON and OFF states at a reading gate voltage of −10 V can be well maintained for more than 10 000 s with a high memory current ratio of 10^2^ with a slight initial fast decay and a subsequently slower decay. The phenomenon was consistent with the model for long‐term trapped charge decay in most polymer/small molecule elements.^[^ ^59^ ^]^ The endurance and retention results indicated that the optimized molecule **1**‐based device is promising in reliable and stable memory for high‐density information storage.

## 3 Conclusion

We have demonstrated an unexpected acid‐promoted intramolecular alkyne cycloisomerization for synthesizing two configurational isomers expanded [9]helicene (**1**) and π‐extended double [4]helicene (**2**). Single‐crystal X‐ray analysis unambiguously revealed their different helical structures and packing modes. Matrix‐assisted laser desorption/ionization time‐of‐flight mass spectrometry (MALDI‐TOF‐MS) and UV–vis–PL revealed that compound **1** shows weaker intermolecular interaction than **2**. CV spectra and DFT calculations show that both of **1** and **2** have the similar electronic structures with wide energy gaps, which further applied as MFG in OFET‐NVMs. Although compounds **1** and **2** have nearly identical HOMO/LUMO energy levels, the molecular geometry showed a significant effect on charge‐trapping behavior. The expanded [9]helicene **1** based device displays a more than threefold wider MW (44.5 V) than that of π‐extended double [4]helicene **2** based device (14.2 V) and a large charge‐trapping density of 1.08 × 10^13 ^cm^−2^. An unprecedented correlation has been established between molecular‐geometry‐induced packing patterns and charge‐trapping behavior. A large ON/OFF ratio (≈10^5^), long‐term charge retention (>10^4^ s), and reliable endurance (800 cycles) are obtained in the optimized **1**‐based device. This study reveals that controlling the geometry of topological molecular nanocarbons plays a crucial role in charge‐trapping OFET‐NVMs, which enables us to understand the structure–property relationships of charge‐trapping molecules and future molecular design for high‐performance organic memory devices.

## 4 Experimental Section

Experimental details of general information, synthesis details, characterizations, theoretical calculations, and device fabrications are described in Supporting Information.

CCDC 2 244 909 (for **1**) and 2 244 910 (for **2**) contain supplementary crystallographic data for this paper. These data can be obtained free of charge from The Cambridge Crystallographic Data Centre via www.ccdc.cam.ac.uk/data_request/cif.

## Conflict of Interest

The authors declare no conflict of interest.

## Supporting information

Supplementary Material

## Data Availability

The data that support the findings of this study are available in the supplementary material of this article.
