# Supplementary material for: Intramolecular Alkyne Aromatization: Unexpected Synthesis of Expanded [9]Helicene and π‐Extended Double [4]Helicene, and Their Molecular Geometry Effect on Transistor Memory
Source: Small Sci. 2023 Jun 29;3(8):2300040. doi: 10.1002/smsc.202300040 (PMC11935940; doi:10.1002/smsc.202300040)
Supplement: Supplementary file 1 — Supplementary Material [file SMSC-3-2300040-s001.pdf]

Supporting Information

Intramolecular Alkyne Aromatization: Unexpected Synthesis of Expanded [9]Helicene and π-Extended Double [4]Helicene, and Their Molecular Geometry Effect on Transistor Memory

Yang Yu, Le Wang, Chang Wang, Fei Liu, Haifeng Ling and Junzhi Liu**

Content

1. Experimental methods……………………………………………………….…...…2

2. Synthesis……………………………………………………………………….……4

3. Optimization of the reaction conditions……………….……………………….....8

4. Comparison of the NMR spectra of compounds **1**, **2** and **4**.………………………...9

5. Proposed mechanism of cycloisomerization.……………………………………...10

6. X-ray diffraction of compound **1** and **2**.……………………………………………11

7. Contact angles…...…………………….…………………………...……………12

8. OFET memory devices and performances………………………………………...13

9. NMR spectra……………………………………………………………………....22

10. High-resolution mass spectrometry………………………………………………30

11. References……………………………………………………………………..36

1. Experimental methods

General Information:

All the solvents and reagents were purchased from commercial suppliers and used without further purification, unless noted otherwise. 4,4'-((4,6-dibromo-1,3-phenylene)bis(ethyne-2,1-diyl))bis(*tert*-butylbenzene)[1] and 4,4,5,5-tetramethyl-2-(phenanthren-3-yl)-1,3,2-dioxaborolane[2] were prepared using literature methods. Pentacene and Polystyrene (PS, weight average molecular weight M_w = 192 000 g/mol) for device fabrication were purchased from Sigma-Aldrich and used without further purification. The preparation details are described as follows.

Characterization:

All NMR spectra were acquired on the Avance DRX Bruker 400, 500 and 600 MHz FTNMR Spectrometer. High resolution mass spectra (ESI) were obtained on a Bruker Q-Tof Maxis II mass spectrometer and a DFS high resolution magnetic sector mass spectrometer. Absorption spectra were measured with a Shimadzu UV-3150 spectrometer, and emission spectra were recorded on a Shimadzu RF-530XPC luminescence spectrometer. Cyclic voltammetric (CV) studies were conducted using a CHI600C in a typical three-electrode cell with a glassy carbon working electrode, a platinum wire counter electrode, and a silver reference electrode. Atomic force microscopy (AFM) measurements were obtained with a NanoScope IIIa AFM at room temperature. Commercial silicon cantilevers with typical spring constants of 21-78 Nm^{-1} were used to operate the AFM in tapping mode.

Theoretical Calculations:

In order to predict the geometrical and electronic properties of the molecules, the Gaussian 16 program package was applied by means of DFT at the level of B3LYP with a 6-311G(d,p) basis set. The molecular orbitals were visualized in GaussView 5.0. Anisotropy of the induced current density (ACID) plots and nucleus-independent chemical shifts (NICS) were used to compare the aromaticity of two structural isomers

by adopting Herges's method[3] and standard gauge invariant atomic orbital (GIAO)[4-6] method at B3LYP functional. The 6-311+G(2d,p) basis set was used for the C and H atoms. All NICS values were acquired from the average of two positions (above and below the plane) of each molecule for reducing the error caused by the curvature of the molecule.

Device fabrication and characterization:

OFET memory devices were fabricated with a top-contact and bottom-gate configuration. A heavily doped *n*-type Si wafer with 90 nm SiO_2 as the gate dielectric was used as the substrate. The substrates were cleaned sequentially in an ultrasonic bath with acetone, ethanol, and deionized water for 10 min each and then transferred into an oven at 120 °C for 30 min after drying using a nitrogen gun. Later, the substrate surface was UV/ozone cleaned for 10 min and transferred to an N_2 filled glovebox. A toluene solution of compound **1** or compound **2** was stirred for 30 min to form a homogeneous solution. All the solution-processed films were fabricated by spin-coating at the spin speed of 2000 rpm for 30 s and then baked at 100 °C on a hot plate for 30 mins to remove the residual solvent. The thickness of the prepared thin films were estimated to be 20 nm. After that, 50 nm thick pentacene was thermally evaporated at a deposition rate of 0.1 Å s^{-1} under the pressure of 5×10^{-4} Pa. Finally, about 100 nm thick Cu was thermally evaporated through a shadow mask to form source and drain electrodes with the channel width W=1500 μm and length L=150 μm. Film thickness was measured by the Bruker Dektak XT stylus profiler. The areal capacitance of the SiO_2/**1** and SiO_2/**2** layers was measured using HIOHI IM3533-01. The electrical characteristics of the memory devices were carried out on a Keithley 2636b System Source Meter. All electrical measurements were carried out under ambient conditions at room temperature.

2. Synthesis

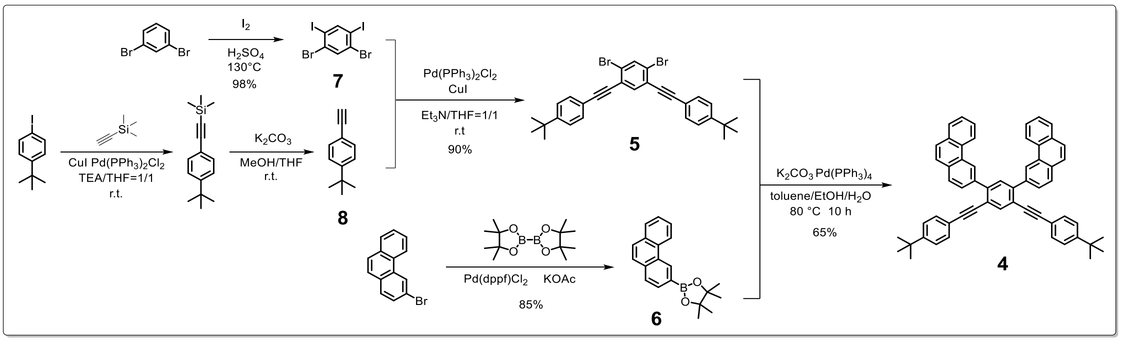

Scheme S1. Schematic illustration of the synthesis of **4**.

The known compounds **5**[1], and **6**[2], **7**[32], **8**[33] were synthesized according to the literature methods without any modification. Compound **8** is also commercially available.

*Synthesis of 3,3'-(4,6-bis((4-(tert-butyl)phenyl)ethynyl)-1,3 phenylene)diphenanthrene (**4**).*

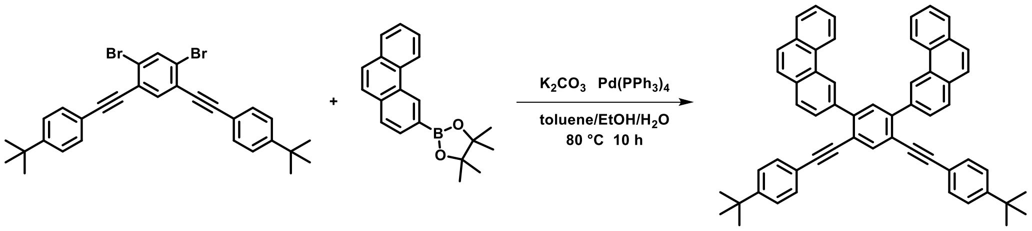

In a 100 mL Schlenk flask, 4,4'-((4,6-dibromo-1,3-phenylene)bis(ethyne-2,1-diyl))bis(*tert*-butylbenzene) (230 mg, 419.43 μmol) and 4,4,5,5-tetramethyl-2-(phenanthren-3-yl)-1,3,2-dioxaborolane (357.25 mg, 1.17 mmol) were charged under the protection of nitrogen. After adding 10 mL toluene, 2.5 mL ethanol and 2.5 mL K_2CO_3 aqueous solution (2 mol/L), the mixture was degassed for 30 mins. $Pd(PPh_3)_4$ (48.47 mg, 41.94 μmol) was added, then the mixture was heated to 90 °C and stirred for 12 hours. The reactant was extracted by 20 mL ethyl acetate (EA) for three times, then dried over magnesium sulfate and the solvent was evaporated in vacuo. The crude product was purified by chromatography on silica gel (hexane/CH_2Cl_2 =8/1) to give the

product **4** as yellow powder (200 mg, 269.18 μmol, yield:64.18%). ^{1}H NMR (500 MHz, CD_2Cl_2) δ 9.19 (s, 2H), 8.79 – 8.77 (m, 2H), 8.12 (s, 1H), 8.10 – 8.04 (m, 4H), 7.96 – 7.94 (m, 3H), 7.86 – 7.82 (m, 4H), 7.65 – 7.61 (m, 4H), 7.29 – 7.25 (m, 8H), 1.27 (s, 18H). ^{13}C NMR (101 MHz, CD_2Cl_2) δ 152.50, 144.03, 138.50, 138.28, 132.87, 132.16, 132.05, 131.75, 130.94, 130.55, 129.18, 128.79, 128.47, 127.97, 127.36, 127.32, 127.12, 125.93, 124.11, 123.41, 121.71, 120.43, 94.12, 88.48, 35.24, 31.40. HRMS (ESI) m/z $[M+H]^+$ Calcd for $C_{52}H_{48}O_4H^+$ 743.3672; Found 743.3646.

Synthesis of molecule ***1*** *and* ***2****. (Condition A)*

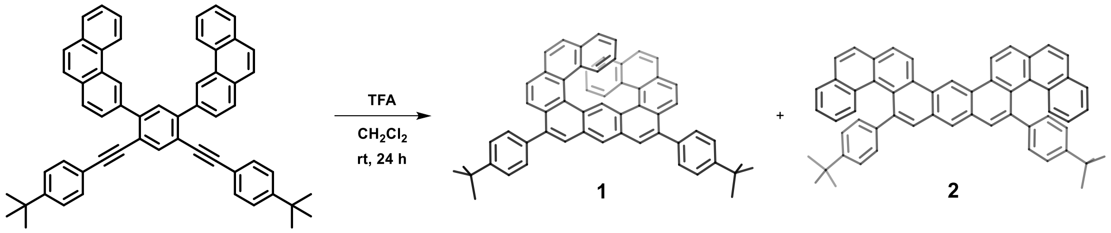

4 (150 mg, 201.88 μmol) was dissolved in 20 mL of dry CH_2Cl_2. To the solution was added 1 mL trifluoroacetic acid at room temperature and stirred for 1 day. After reaction, the solution was added to 10% $NaHCO_3$ solution and extracted by DCM for three times. The organic phase was dried over magnesium sulfate and the solvent was evaporated in *vacuo*. The crude product was purified by flash chromatography on silica gel (hexane/CH_2Cl_2 =6/1) to give the product **1** as brownness powder (40.5 mg, 54.51 μmol, yield:27%) and **2** as yellow powder (76.5 mg, 102.96 μmol, yield:51.0%) **Product 1**: ^{1}H NMR (600 MHz, CD2Cl2) δ 10.93 (s, 1H), 8.41 (s, 1H), 8.18 (d, *J* = 8.4 Hz, 2H), 7.94 (s, 2H), 7.85 (d, *J* = 8.3 Hz, 2H), 7.69 (d, *J* = 5.4 Hz, 2H), 7.68 (d, *J* = 5.5 Hz, 2H), 7.64 (s, 2H), 7.62 (d, *J* = 1.9 Hz, 2H), 7.60 (br, 8H), 6.94 (t, 2H), 6.41 (t, 2H), 1.45 (s, 18H). ^{13}C NMR (151 MHz, CD_2Cl_2) δ 151.22, 139.34, 138.44, 132.60, 132.34, 131.91, 131.09, 130.68, 130.51, 129.16, 128.61, 128.27, 127.77, 127.60, 127.37, 127.05, 126.95, 126.37, 126.16, 126.05, 126.00, 125.69, 123.82, 35.16, 31.76. HRMS (EI) m/z $[M]^+$ Calcd for $C_{52}H_{48}O_4^+$ 742.3600; Found 742.3585. **Product 2**: ^{1}H NMR (600 MHz, 353K, $C_2D_2Cl_4$) δ: 10.25-10.24 (d, *J* = 6.0 Hz, 1H), 9.30-9.24 (dd, 2H), 8.70 (s, 1H),

8.26-8.24 (m, 4H), 8.02-8.00 (t, 2H), 7.97-7.95 (d, 2H), 7.90-7.88 (d, 2H), 7.74-7.33 (d, 2H), 7.26-7.24 (t, 2H), 7.24-6.99 (m, 8H), 6.99-6.97 (t, 2H), 1.26 (s, 18H). ^{13}C NMR (151 MHz, $C_2D_2Cl_4$) δ: 149.09, 140.01, 139.06, 131.78, 131.35, 131.19, 130.77, 130.20, 129.34, 128.81, 128.67, 128.43, 128.12, 127.82, 127.41, 126.86, 126.40, 125.79, 125.33, 124.98, 124.37, 123.88, 121.33, 121.24, 117.49, 117.19, 99.29. 33.86, 30.88. HRMS (ESI) m/z $[M+H]^+$ Calcd for $C_{52}H_{48}O_4H^+$ 743.3672; Found 743.3649.

*Synthesis of 7,11-bis(4-(tert-butyl)phenyl)-8,10-diiodonaphtho[2,1-c]phenanthro[4,3-m]tetraphene (**3**).*

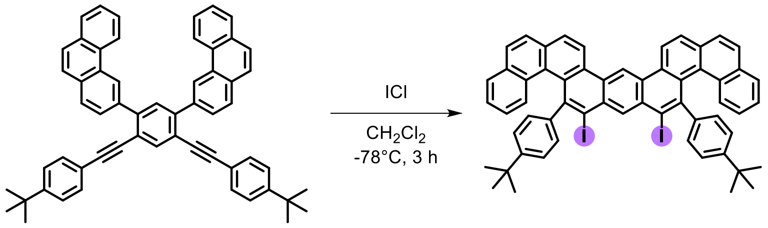

A solution of **4** (50 mg, 67.29 µmol) in anhydrous DCM (10 ml) was purged with N_2. After the solution was cooled to -78 °C, a 1 M solution of iodine monochloride in DCM (168 µL, 2.5 equiv.) was added dropwise under N_2 atmosphere. The reaction was stirred for 3 h and quenched with saturated sodium sulfite solution. The reaction mixture was diluted in 20 ml DCM and the organic layer was washed with a saturated sodium sulfite solution (3×10 mL) and dried over magnesium sulfate and the solvent was evaporated in vacuo. The crude product was purified by a flash chromatography on silica gel (hexane/CH_2Cl_2 =4/1) to give the product **3** as yellow powder (54 mg, 54.28 µmol, yield: 80.6%). The product shows poor solubility, so we can only acquire the ^{1}H-NMR and high-resolution Maldi-tof-ms. The crude product can directly go to the next step without purification. ^{1}H NMR (400 MHz, 298K, CD_2Cl_2) δ: 9.96 (d, 1H), 9.82 (d, 1H), 9.08 (dd, 2H), 8.30 (br, 2H), 8.20 (dd, 2H), 8.11 (d, 2H), 7.84 (dd, 2H), 7.77 (d, 2H), 7.61 (d, 2H), 7.30 (br, 2H), 7.28 – 7.22 (t, 2H), 7.16 (t, 2H), 6.55 (br, 2H), 5.81 (br, 2H), 1.14 (s, 18H). HRMS (Maldi-tof-ms) m/z $[M]^+$ Calcd for $C_{58}H_{44}I_2^+$ 994.1532; Found 994.1923.

*Synthesis of molecule **2** from **3**. (Condition B)*

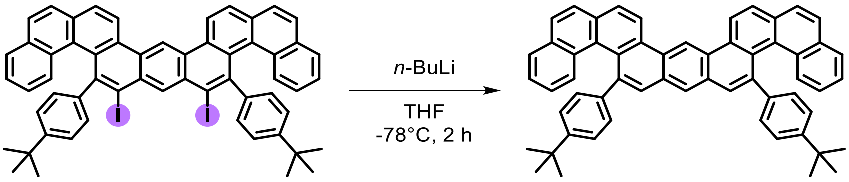

A solution of **3** (crude product, 50 mg, 50.26 mmol) in anhydrous THF (25 mL) was degassed with N_2 for 30 min. After the solution was cooled to –78 °C, a 1.6 M solution of *n*-BuLi (79 μL, 126 μmol) was added dropwise to the solution under N_2 atmosphere. The reaction was stirred for 2 h and quenched with methanol (5 mL). The organic solvent was removed in vacuo, and the residue was washed with water and MeOH to afford **2** (35.5 mg, 95%) as a yellow powder. The NMR and HRMS data are the same with condition A.

3. Optimization of the reaction conditions.

Table S1. Optimization of cycloisomerization reaction conditions.[a]

Entry	Catalyst	Ligand	Solvent	T(°C)	Products	Yield (%)[b]
1	$PtCl_2$	-	Toluene	80-100	-	n.r.[c]
2	$PtCl_2$	$P(Ph)_3$	Toluene	80-100	-	n.r.[c]
3	$PtCl_2$	$P(C_6F_5)_3$	Toluene	80-100	**2** and u.p.[d]	(**2**) trace
4	$InCl_3$	-	Toluene	100	**2** and u.p.[d]	(**2**) trace
5	AgOTf	-	Toluene	80	-	n.r.[c]
6	TfOH	-	DCM	rt	**1, 2** and u.p.[d]	(**1**)8, (**2**)21
7	TFA	-	DCM	rt	**1** and **2**	(**1**)27, (**2**)51
8	ICl	-	DCM	-78	**3**	(**3**)80

[a)] 50 mg substrate in 20 mL dry solvent under N2 atmosphere. [b)] Isolated yield. [c)] No reaction. [d)] Unresolved products (u.p.).

At first, transition-metal Lewis acid catalysts were carried out (Table S1). In the presence of $PtCl_2$ or AgOTf, compound **4** was not reacted under 80 to 100 °C by oil-bath heating or using microwave reactor (Table S1, entries 1 and 5). Accordingly, we optimized reaction conditions using the $PtCl_2$ as the catalyst for the alkyne aromatization (Table S1, entries 2-4), and a trace amount of **2** was obtained. Then ICl-induced cyclization was performed. Iodine substituted intermediate **3** was obtained in good yield (80%) by treatment of **4** with ICl under -78°C. In other ways, Brønsted acid was also investigated for activating the alkyne aromatization of **4**. Upon treatment of **4** with triflic acid (TfOH) at room temperature, compound **2** (yield 21%) and another blue light product **1** was obtained (Table S1, entry 6). Finally, the reaction was optimized by treatment of **4** with excess trifluoroacetic acid (TFA), the cycloisomerization reaction smoothly occurred. The yields of compounds **1** and **2** were 27% and 51%, respectively (Table S1, entry 7).

4. Comparison of the NMR spectra of compounds 1, 2 and 4.

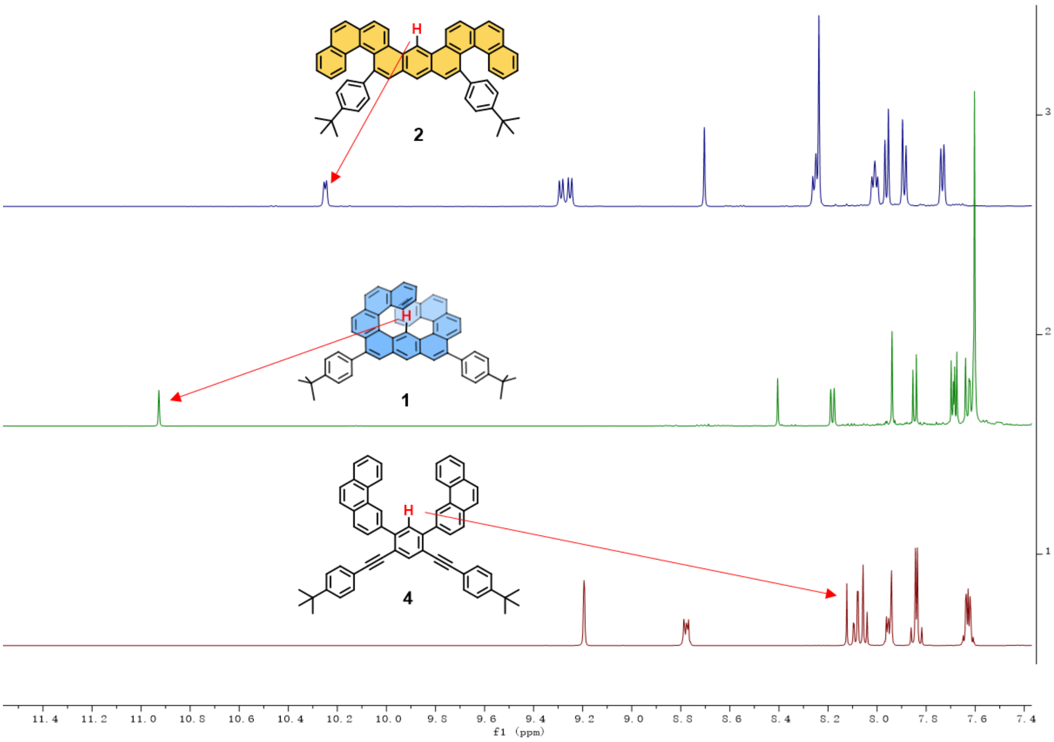

Figure S1. Comparison of the ^{1}H-NMR spectra of compounds **1**, **2** and **4**.

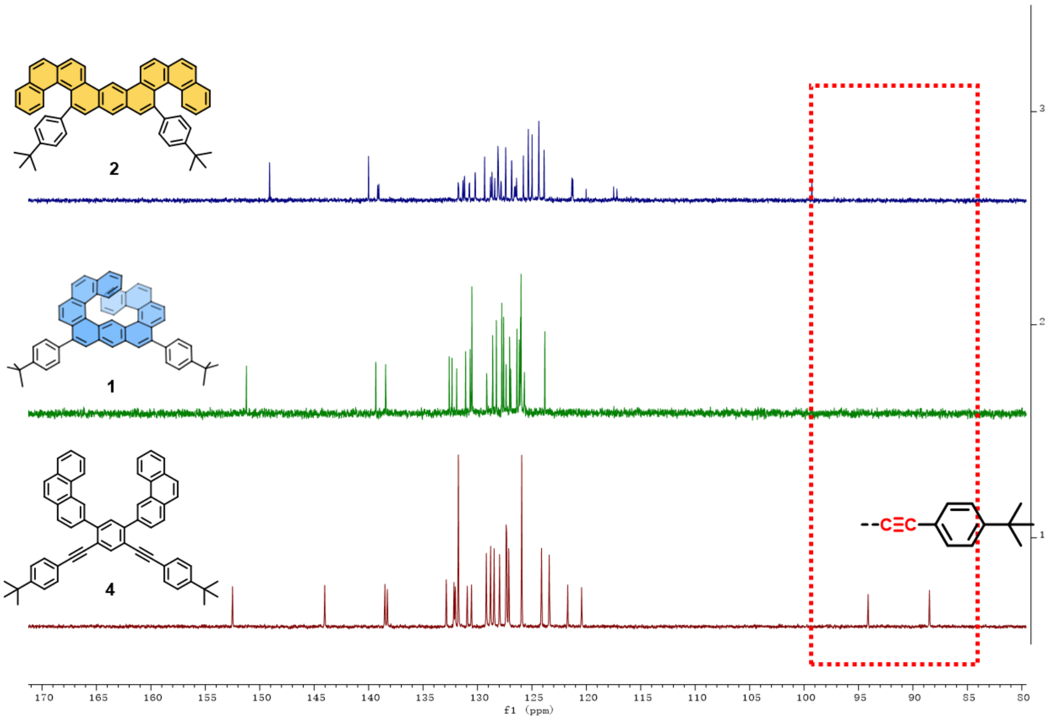

Figure S2. Comparison of the ^{13}C-NMR spectra of compounds **1**, **2** and **4**.

5. Proposed mechanism of cycloisomerization.

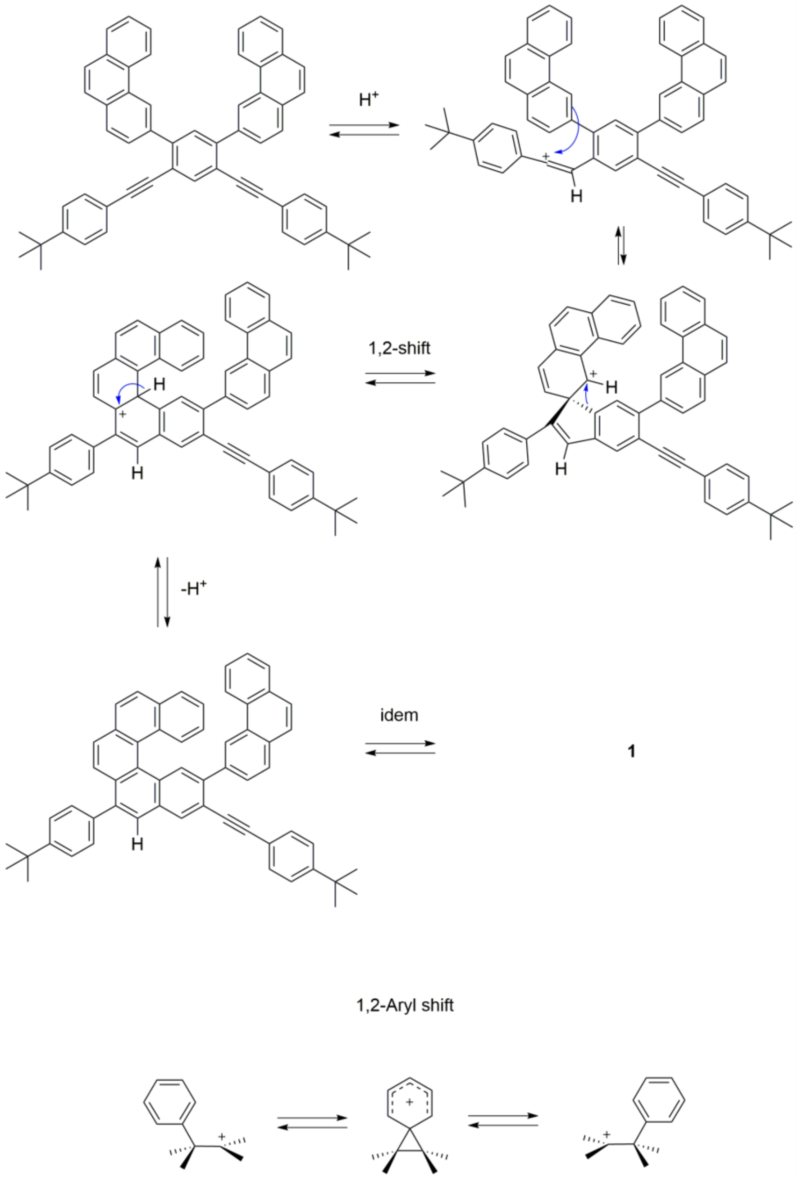

Scheme S2. The proposed mechanism of TFA-induced cyclization for the synthesis of **1**.

6. X-ray diffraction of compound 1 and 2.

The single crystal of **1** and **2** were grown by dilution crystallization. Specifically, dichloromethane (DCM) and hexane were chosen as the good solvent and poor solvent, respectivily. Through slow diffusion of hexane into DCM, the single crystals were obtained after a period of time. The X-ray crystallographic coordinates for structures reported in this article have been deposited at the Cambridge Crystallographic Data Centre (CCDC), under deposition number, CCDC 2244909 (for **1**) and 2244910 (for **2**). These data can be obtained free of charge from CCDC via https://www.ccdc.cam.ac.uk/structures/.

Table S2. the X-ray diffraction data of **1** and **2**.

	**1**	**2**
CCDC No.	2244909	2244910
Moiety formula	$C_{58}H_{46}$	$C_{58}H_{46}$
Formula weight	742.95	742.95
Temperature, K	193	99.99(10)
Crystal size, mm^3	0.1*0.12*0.13	0.04*0.05*0.2
Crystal system	triclinic	monoclinic
space group	P-1	P21/c
a, Å	12.5236(18)	25.7600(18)
b, Å	14.9441(17)	8.2298(4)
c, Å	15.767(2)	40.031(2)
α, deg	86.675(5)	90
β, deg	88.787(5)	100.988(6)
γ, deg	68.893(4)	90
V, $Å^3$	2748.3(6)	8331.1(9)
Z	2	4
Dcalcd., $g \cdot cm^{-3}$	0.898	1.185
F(000)	788	3152
Radiation	Mo Kα (λ = 0.71073)	Cu Kα (λ = 1.54184)
μ, mm^{-1}	0.051	0.504
2Θ range for data collection, °	4.342 to 50.694	5.142 to 131.582
Index ranges	-15<h<15, -17<k<17, -2<l<18	-30<h<21, -9<k<3, -45<l<47
No. of collected reflections	10031	47138
no. of unique ref. (R_{int})	5512(0.0016)	8612(0.1040)
Data/restraints/parameters	5512/78/591	8612/36/1057
R_1, wR_2 [obs I>2σ (I)]	0.0841, 0.2242	0.1165, 0.2738
R_1, wR_2 (all data)	0.1286, 0.2664	0.1672, 0.3060
Largest diff. peak/hole, $e \cdot Å^{-3}$	0.288/-0.318	0.743/-0.375
Goodness-of-fit on F^2	0.985	1.052

7. Contact angles

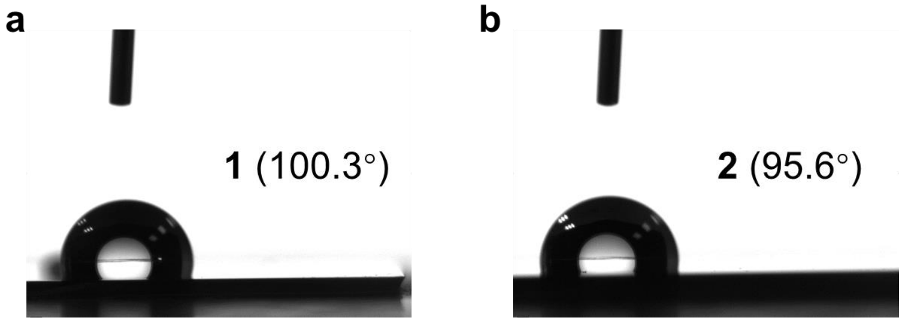

Figure S3. Water contact angles of a) **1**-based and b) **2**-based films surface. Photographs of a water droplet on a) pure **1**-based film and b) pure **2**-based film, respectively.

8. OFET memory devices and performances

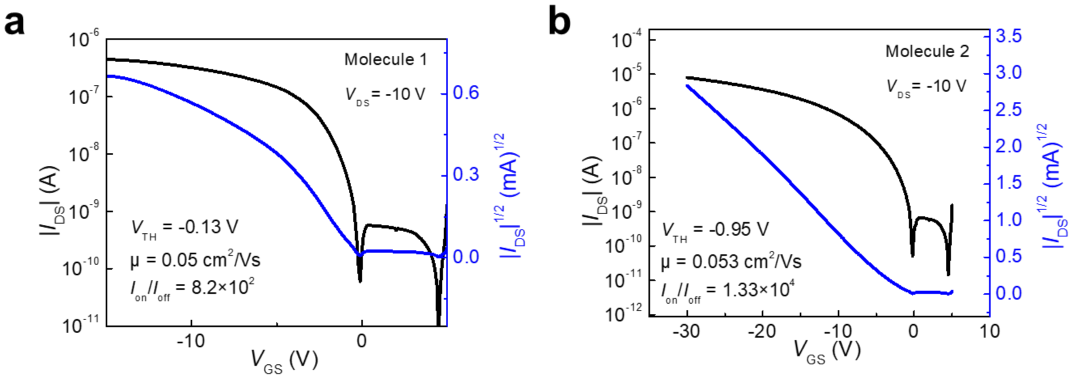

Figure S4. The transfer curves of the OFET-NVM devices with a) pure **1** and b) pure **2**-based charge trapping layers.

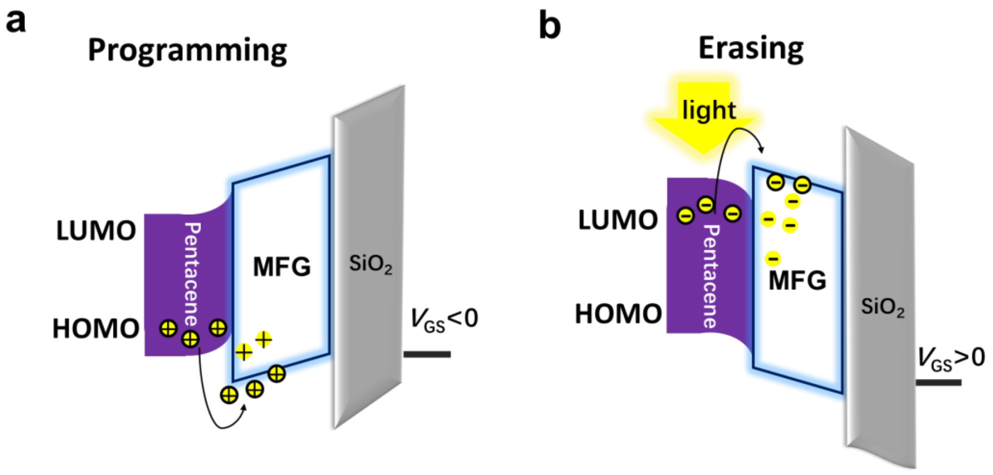

Figure S5. Illustration the proposed mechanism and energy band diagrams of pentacene-based OFET memory device with MFG trapping layer. Hole trapping process: (a) programming with a negative gate bias under dark, (b) erasing under light illumination or positive gate bias.

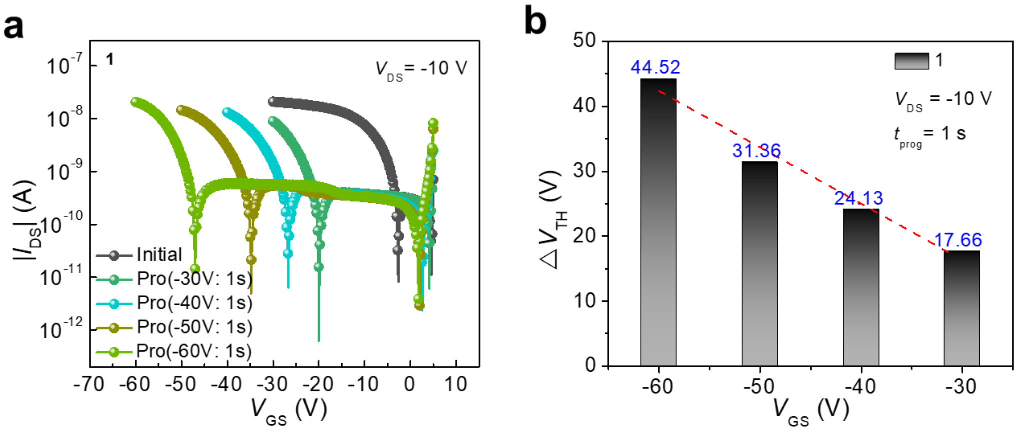

Figure S6. a) Transfer characteristics of **1**-based device operated under various negative programming bias (V_{GS} = 0, -30, -40, -50, -60 V). Memory windows (ΔV_{TH}) and linear fitting curve under different V_{GS}.

Table S3. Transistor and memory performances of OFET-NVMs with the molecule **1** and **2** as MFGs.

MFGs	μ [cm^2 V^{-1} s^{-1}]	V_{TH} [V]	I_{ON} / I_{OFF}	Hole Window [V]	Electron Window [V]
**1**	0.05	-0.13	8.2×10^2	44.5	n.a.
**2**	0.053	-0.95	1.33×10^4	14.2	n.a.

Table S4. Summary of key parameters of our memory device compared with reported state-of-the-art organic small molecules.

**Charge trapping materials & Device structure**	**Mobility [cm^2/Vs]**	**I_{ON}/I_{OFF}**	**Memory window [V]**	**MWR**	**Δn (cm^{-2})**	**Multi levels**	**Endurance cycles**	**Retention Time (s)**	**References**
Si/SiO_2/**M-C10**/P5/Au	2.74	5×10^6	/	/	/	2	1000	2×10^4	[7]
Si/SiO_2/**HHTP/PMMA**/P5/Au	0.05	10^5	28	70%	5.43×10^{12}	2	300	10^4	[8]
Si/SiO2/**o-MeO-DMBI**/PTDPPTFT4/Au	0.004	10^4-10^5	47	47%	2.94×10^{12}	2	1000	6×10^4	[9]
Si/SiO_2/**DCNSFX/P5**/Au	0.003	~10^4	39.3	24.5%	~0.8×10^{12}	3	250	10^4	[10]
Si/SiO_2/**TPA(PDAF)$_3$/P5**/Au	0.053	3.96×10^4	63.2	63.2%	4.55×10^{12}	2	100	10^4	[11]
Si/SiO_2/**Li^+@C_{60}/CYTOP**/P5/Au	0.84	2.18×10^5	32	21.3%	6.91×10^{12}		100	5×10^4	[12]
Si/SiO_2/**CuPc-PS_4**/P5/Au	0.41	10^8	21	42%	2.44×10^{12}	2	100	10^4	[13]
Si/SiO_2/**CuSP1**/P5/Au	0.02	10^6	66	66%	3.55×10^{12}	2	100	10^4	[14]
ITO/HfO_2/**CuPc/C_{60}**/P5/Au	0.012	8.3×10^3	4.4	44%	6.8×10^{11} (h)<br>1.6×10^{12} (e)	2	500	10^4	[15]
Si/SiO_2/**WG_3**/P5/Au	0.33	10^6	45	45%	2.97×10^{12}	2	150	10^4	[16]
Si/SiO2/PVP/P5/P13/P5/Au	0.23	10^4	60	60%	4.35×10^{12}	4	1000	10^4	[17]
Si/SiO_2/**PyPN**/P5/Au	0.004	~10^4	48.43	60.5%	3.2×10^{12}	2	150	10^4	[18]

Si/*h*TSO/**DDTT-SBT-14**/Au	0.25	1.3×10^3	2.18	72%	/	2	150	10^4	[19]
Si/SiO_2/OTS/**BBTNDT**/Ag	7.7	> 10^6	59.7	49.75	/	2	/	10^4	[20]
Si/SiO_2/$(PTPMA)_3$**/TIPS-PEN/**BPEPTCDI/Au	0.0027	5.66×10^5	28	28%	1.48×10^{12}	2	200	10^4	[21]
Si/SiO_2/PS/**TIPS-PEN/N2200**/Au	0.12	10^7	66	55%	/	4	100	10^4	[22]
Si/SiO_2/**PDI&DNTT**/Au	0.004	10^5	7.85	-	/	2	400	10^4	[23]
Si/SiO_2/**PCBM@PMMA**/P5/Au	/	/	22.1	36.8%	/	4	800	1.2×10^4	[24]
Al/CYTOP/ **TIPS-PEN@PS**/DPP-DTT/Au	0.3	10^6	15	25%	/	4	1000	10^4	[25]
Si/SiO_2/***syn*-B2IPIO@PS/**TIPS-PEN/Ag	0.058	10^5	59.2	59.2%	3.54×10^{12}	2	100	10^4	[26]
Si/SiO_2/**PyDI@PS/**TIPS-PEN/Ag	/	10^3	34	42.5%	1.98×10^{12}	2	400	10^4	[27]
Si/SiO_2/**$[DBTDP]_2Cu_2I_2$@PS**/P5/Au	0.75	10^5	$25^{(h)}$	$31.25\%^{(h)}$	3.4×10^{13}	2	35	10^4	[28]
PEN/P(VDF-TrFE-CFE)/TCC/**C60NPs**/P5/Cu	0.2	10^3	6.5	13%	/	2	220	10^4	[29]
Si/SiO_2/**$TTP\text{-}OCH_3$**/P5/Au	0.0014	3.5×10^4	50	50%	3.3×10^{12}	2	35	10^4	[30]
Si/HfO_2/**F-QD**/P5/Au	0.21	> 10^5	~30	75%	0.23×10^{12}	2	20	10^4	[31]
**Si/SiO_2/1/P5/Cu**	0.05	8.2×10^2	44.5	74%	1.08×10^{13}	4	-	10^4	**This work**

P5 is short for Pentacene. The memory window ratio (MWR) and charge trapping density (Δn) is taken or calculated from values of citations. “h” and “e” represent hole and electron, respectively.

Calculation method as follows:

Charge trapping density: $\Delta n = \frac{\Delta V_{\mathrm{th}} \cdot C_{\mathrm{i}}}{e}$

Memory window ratio: $MWR = \frac{\Delta V_{\mathrm{th}}}{V_{\mathrm{GS}}} \times 100\%$

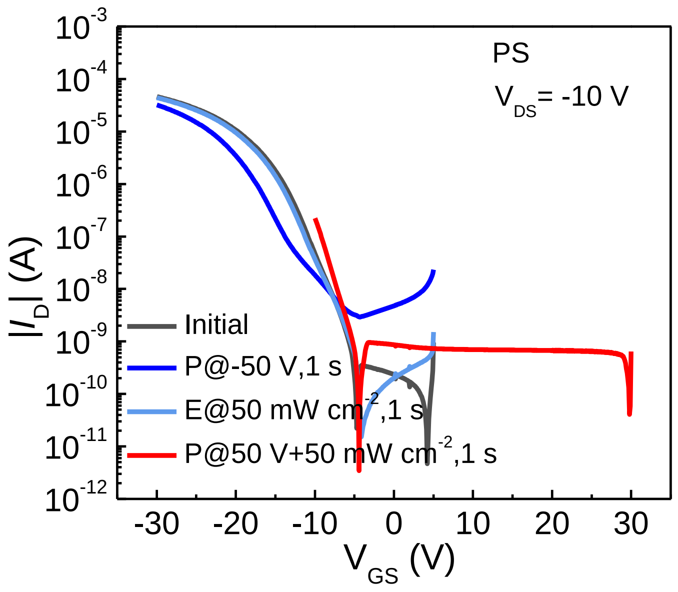

Figure S7. The electrical characteristics of an only PS-based memory device under negative gate bias, and positive gate bias with the assistance of light illumination.

As seen in Figure S7, there was almost no negative memory window when applied gate bias of -50 V, nor a positive memory window when applied gate bias of 50 V with the assistance of high-density light illumination (50 mW/cm^2). In short, the memory performance was mainly determined by the intrinsic properties of the molecules rather than the PS layer or SiO_2 layer.

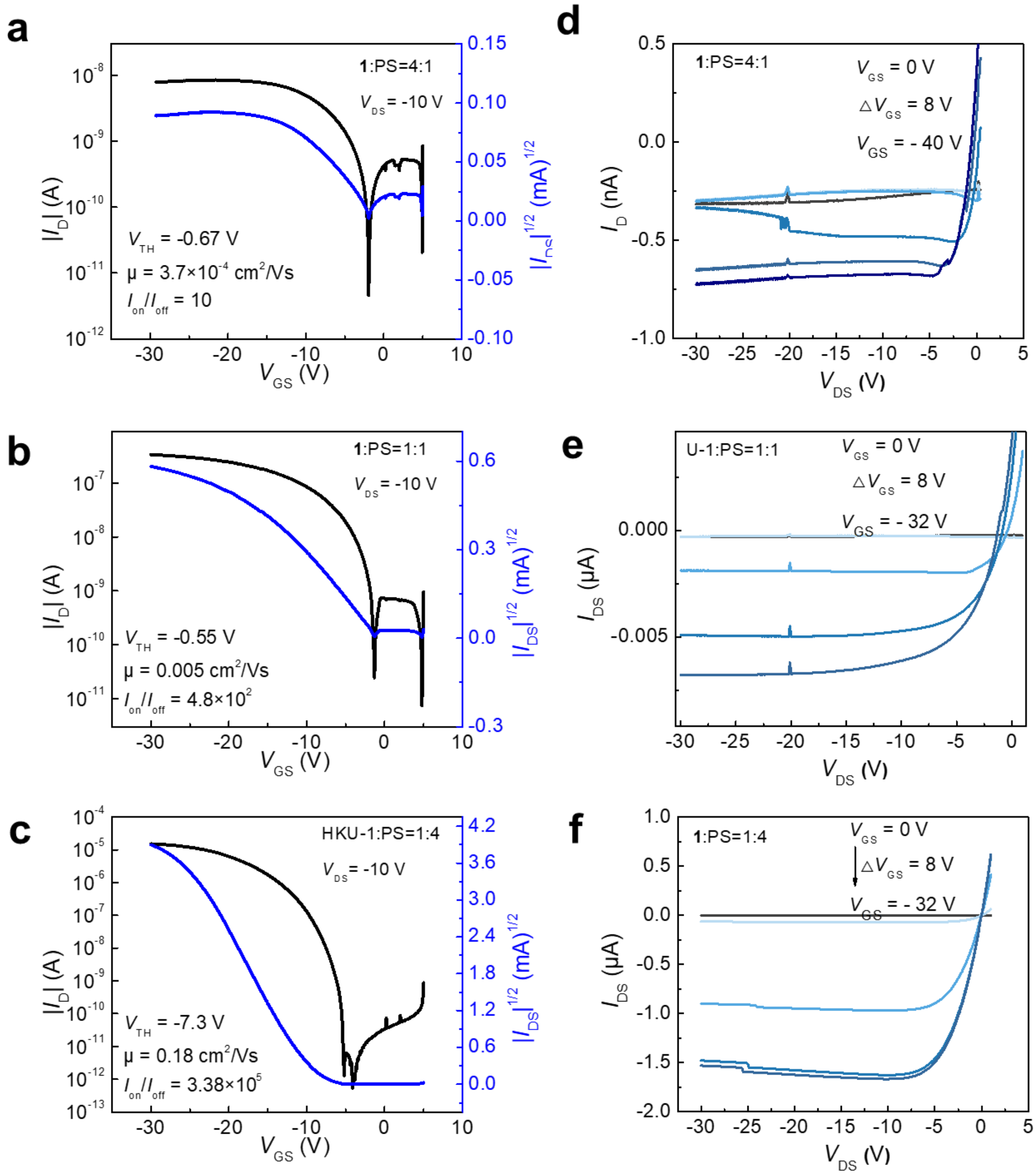

Figure S8. a-c) The transfer curves and d-f) output curves of the OFET-NVM devices with **1**:PS = 4:1, **1**:PS = 1:1, and **1**:PS = 1:4 based charge trapping layers.

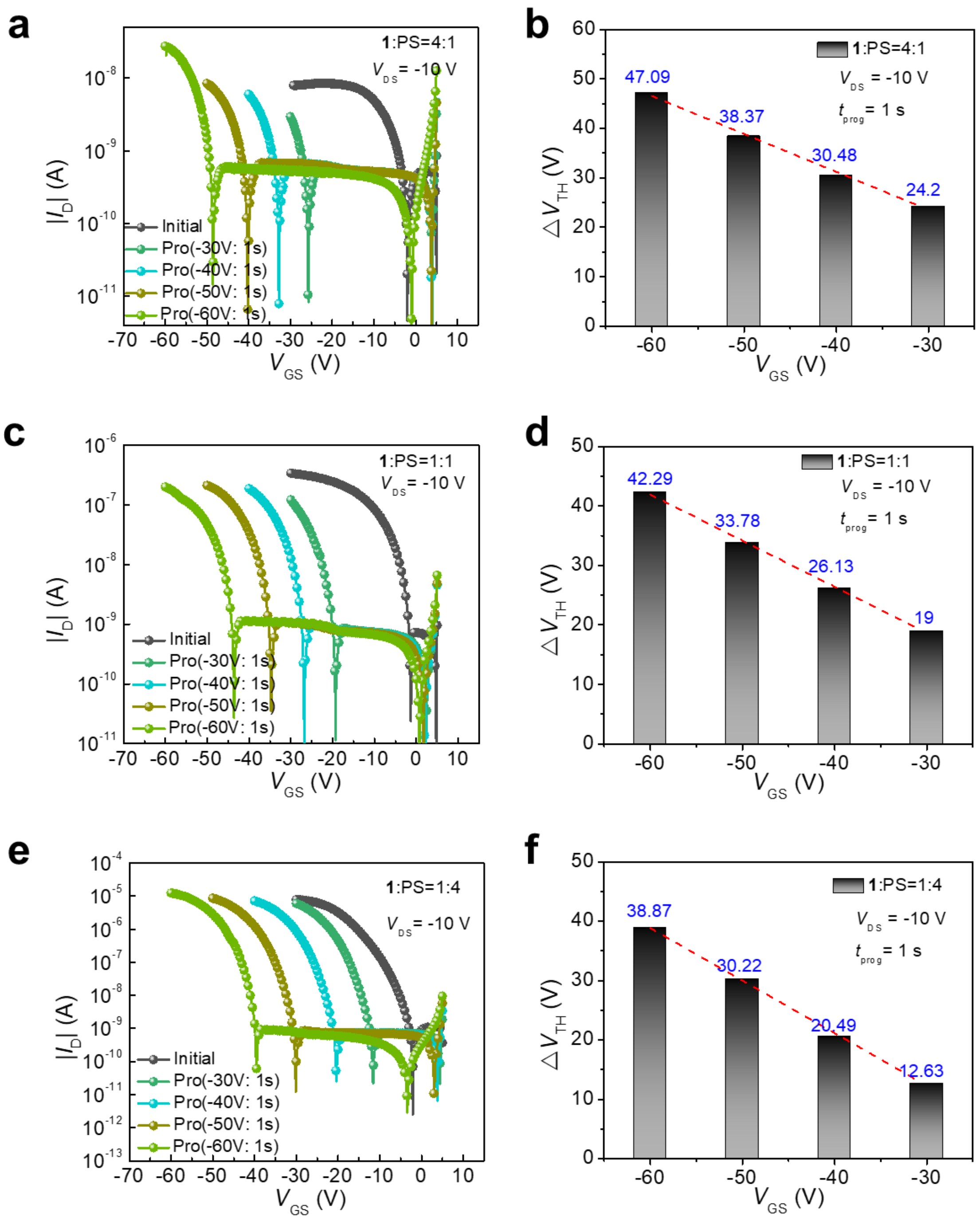

Figure S9. The memory characteristics of a, b) **1**:PS = 4:1, c, d) **1**:PS = 1:1, and e, f) **1**:PS = 1:4-based blend CTLs. All the devices show a stepwise increased ΔV_{TH} with enhanced negative programming bias.

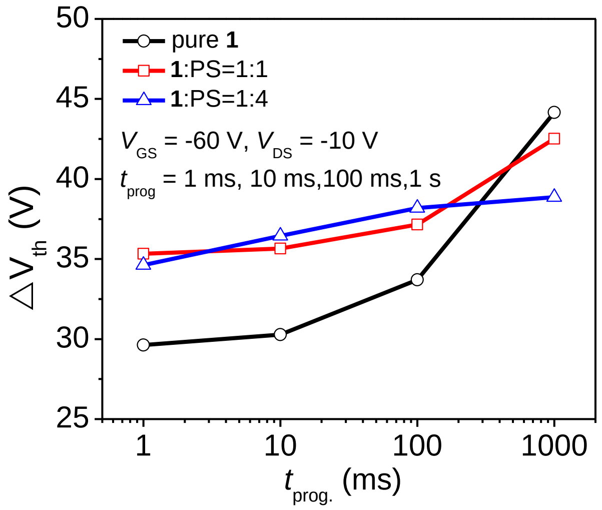

Figure S10. ΔV_{TH} as the function of programming time (t_{prog}) of the OFET-NVM devices with pure **1**, **1**:PS = 1:1, and **1**:PS = 1:4 based CTLs.

9. NMR spectra

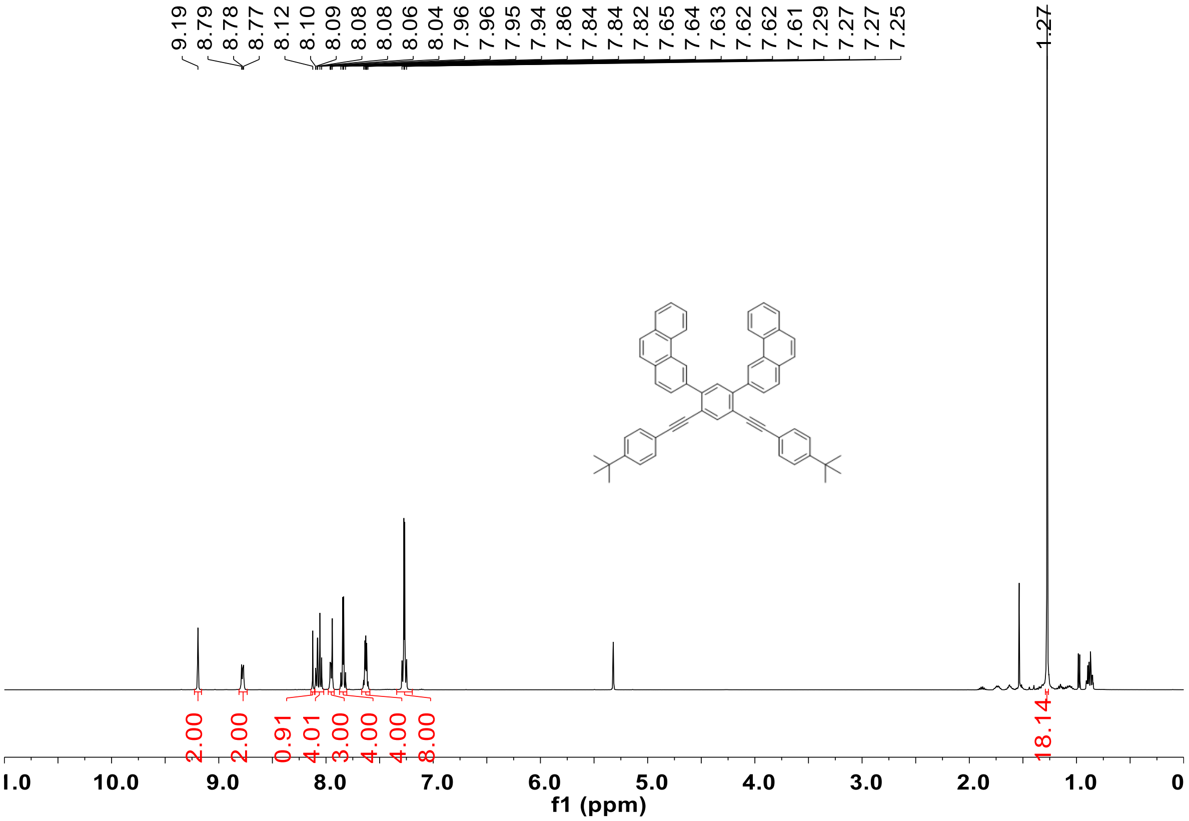

Figure S11. The ^{1}H NMR spectrum (400 MHz) of **4** in CD_2Cl_2 at room temperature.

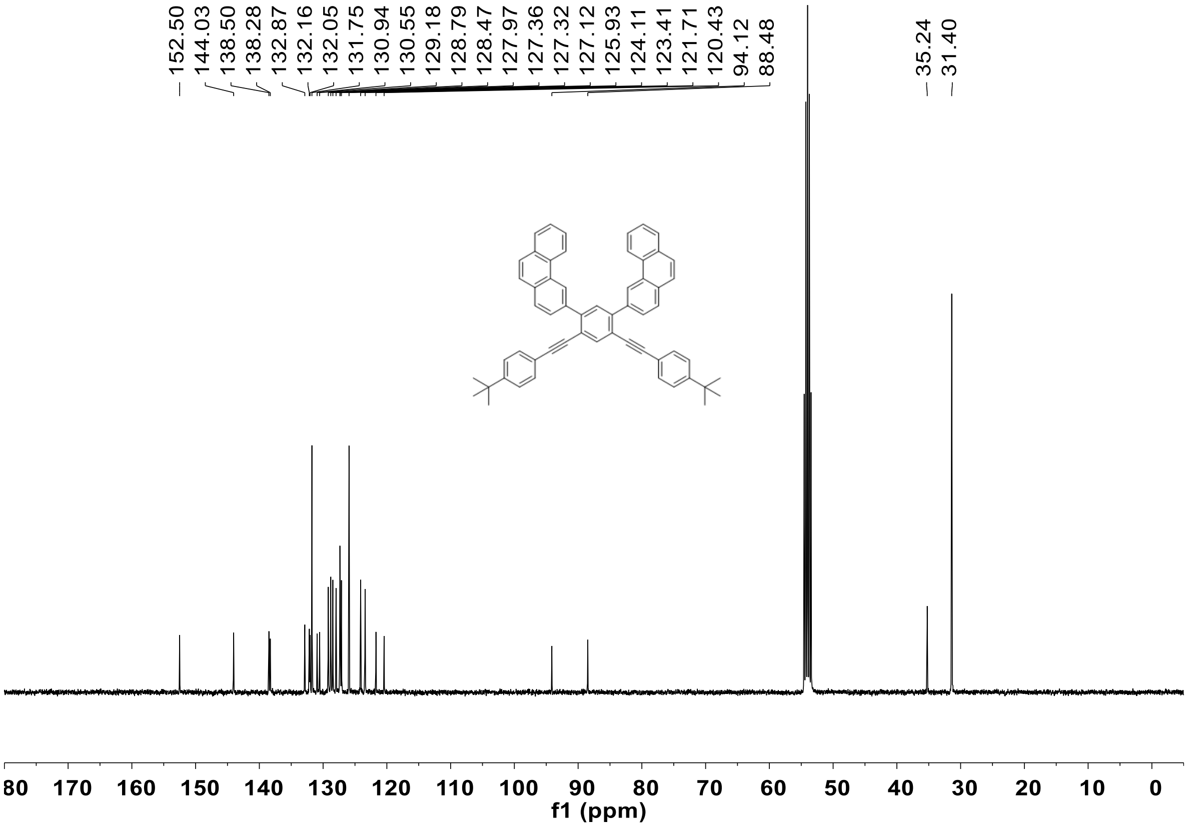

Figure S12. The ^{13}C NMR spectrum (101 MHz) of **4** in CD_2Cl_2 at room temperature.

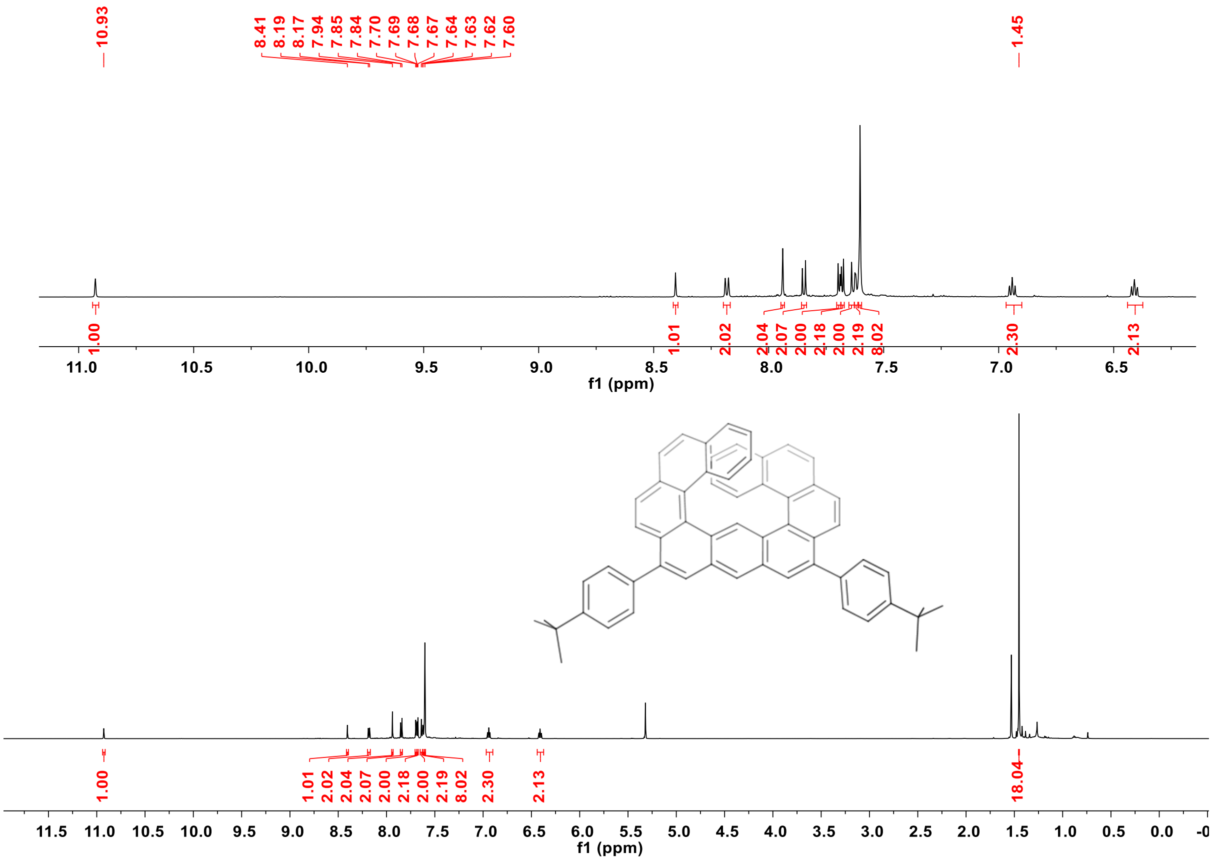

Figure S13. The ^{1}H NMR spectrum (600 MHz) of **1** in $CDCl_2$ at room temperature.

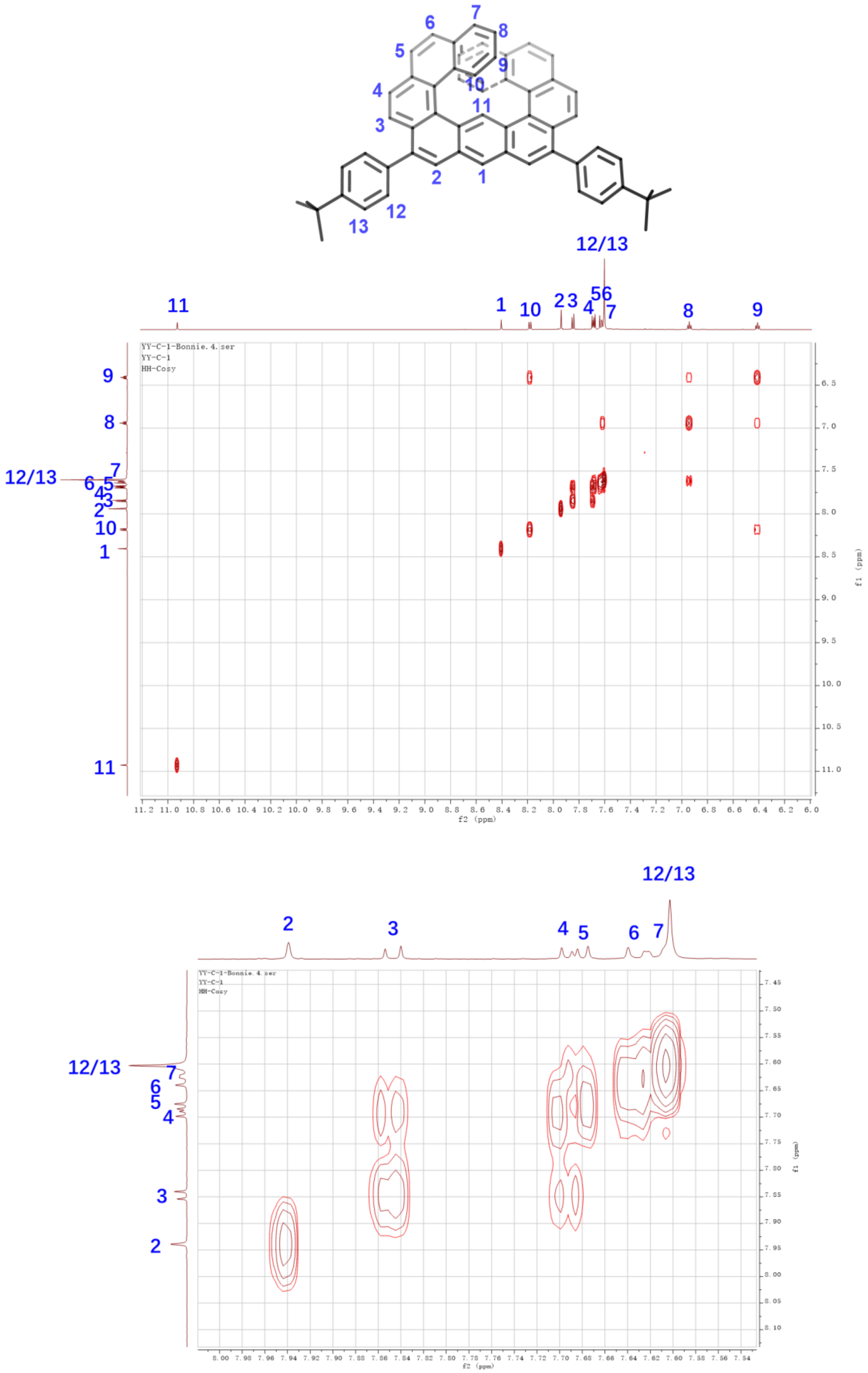

Figure S14. The $^1H/^1H$ COSY spectrum of **1** in $CDCl_2$ at room temperature.

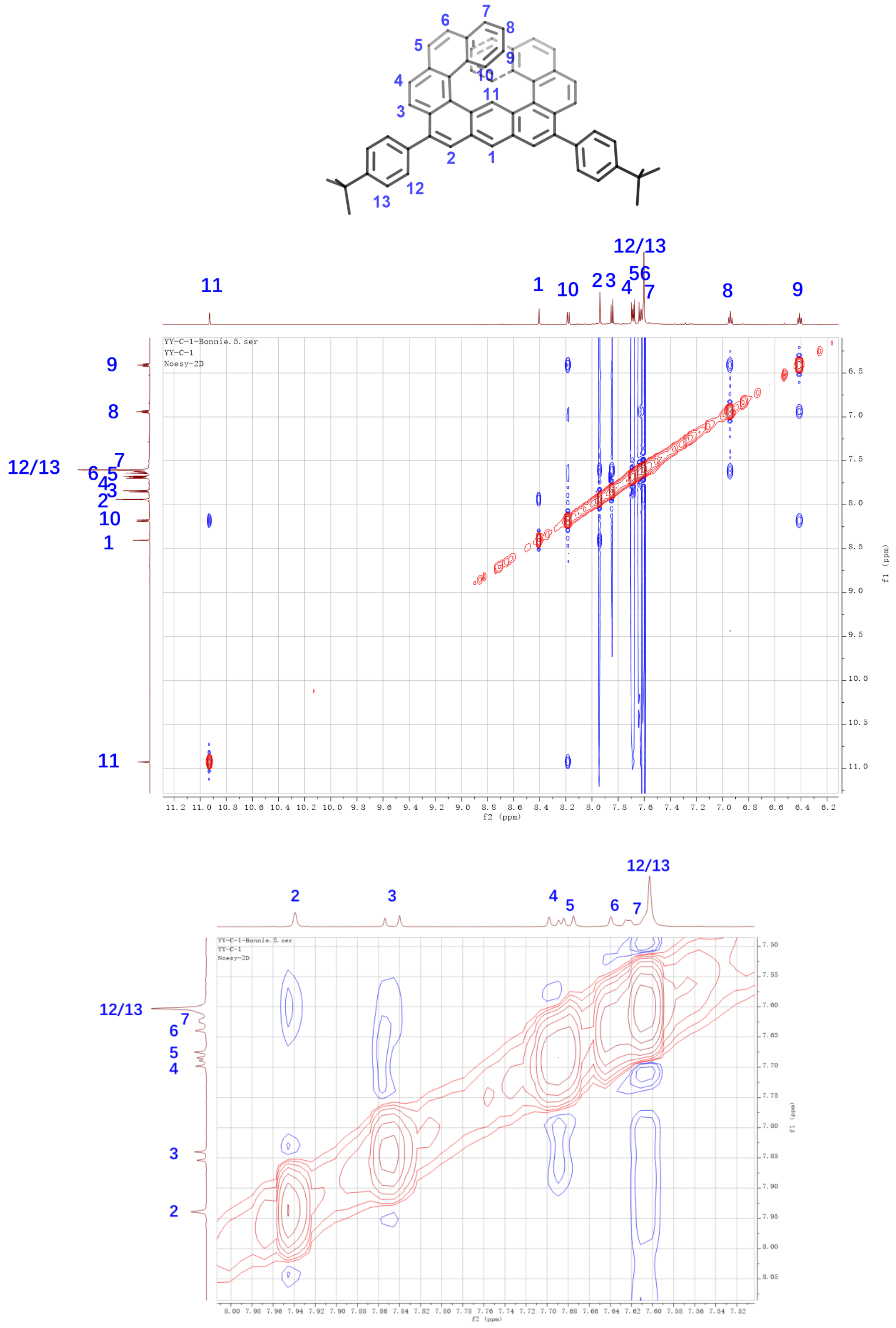

Figure S15. The NOESY spectrum of **1** in $CDCl_2$ at room temperature.

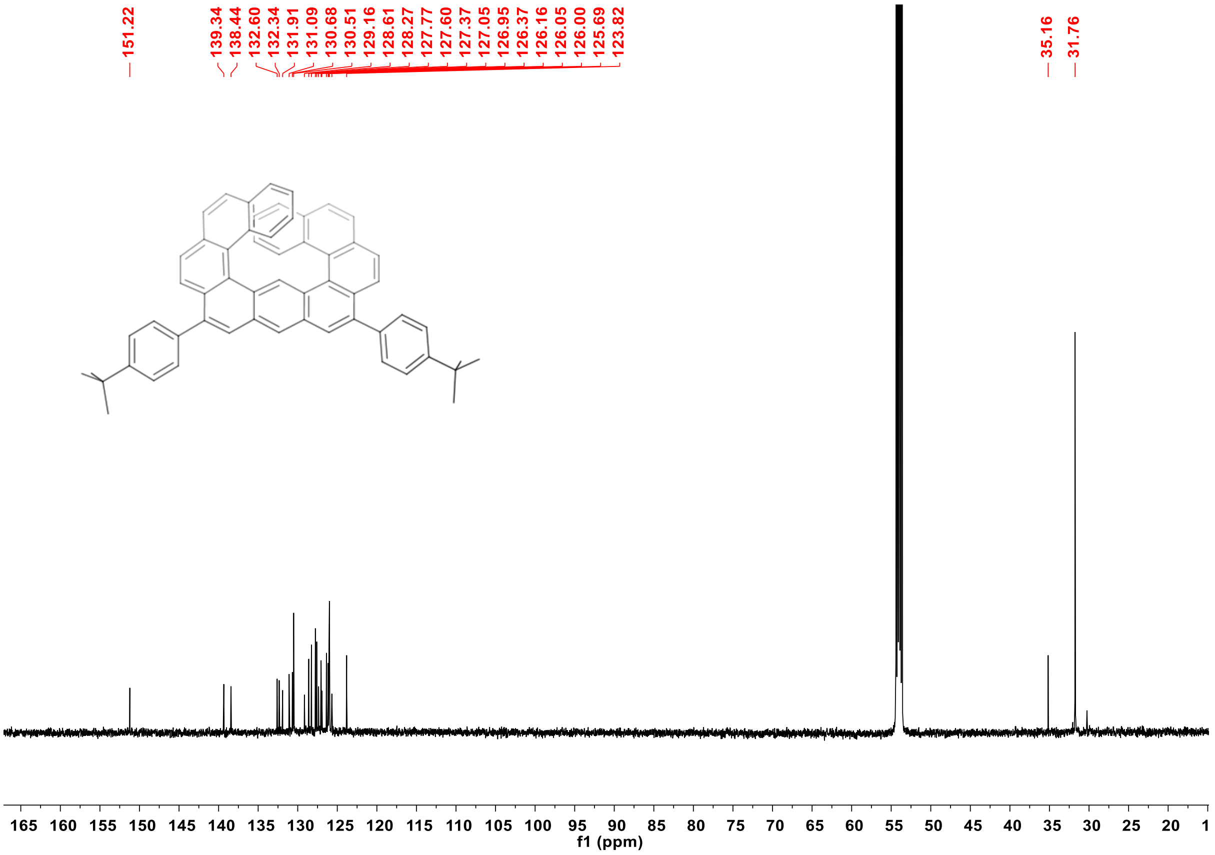

Figure S16. The ^{13}C NMR spectrum (600 MHz) of **1** in $CDCl_2$ at room temperature.

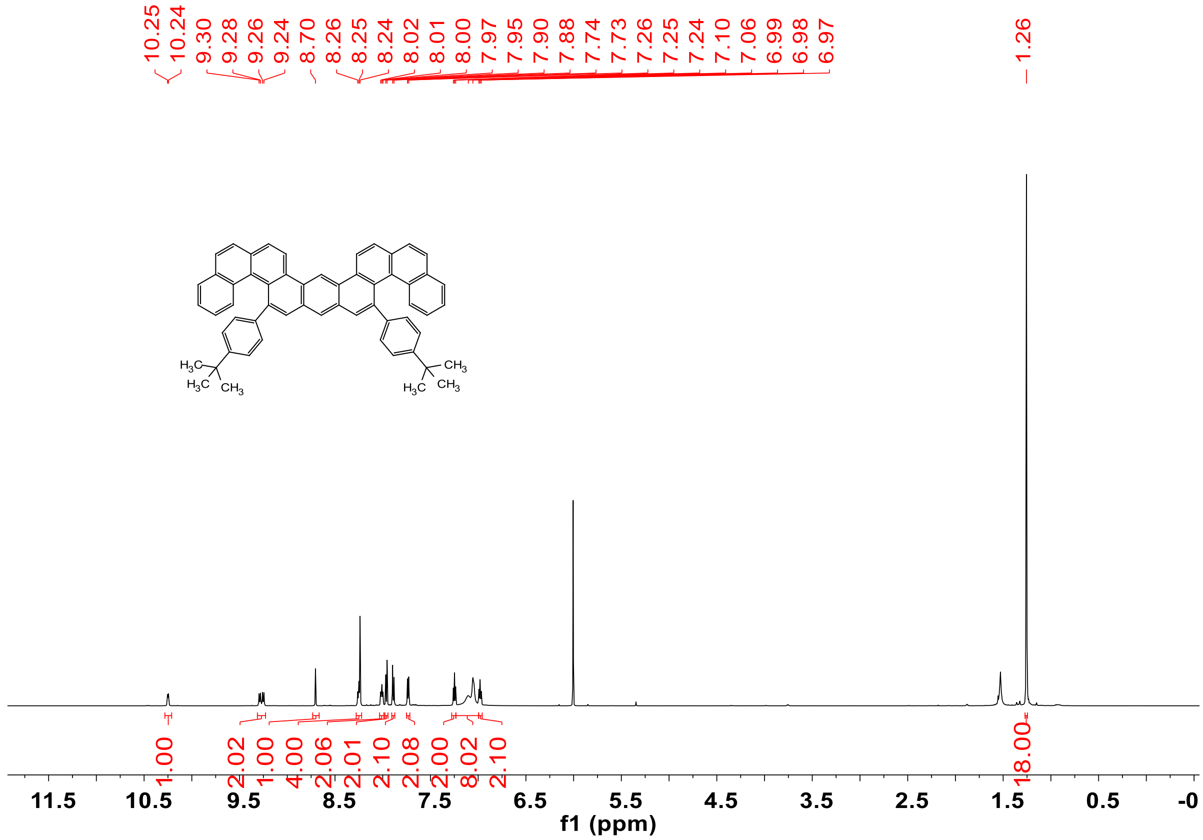

Figure S17. The ^{1}H NMR spectrum (500 MHz) of **C-2** in TCE-d_2 at 353K.

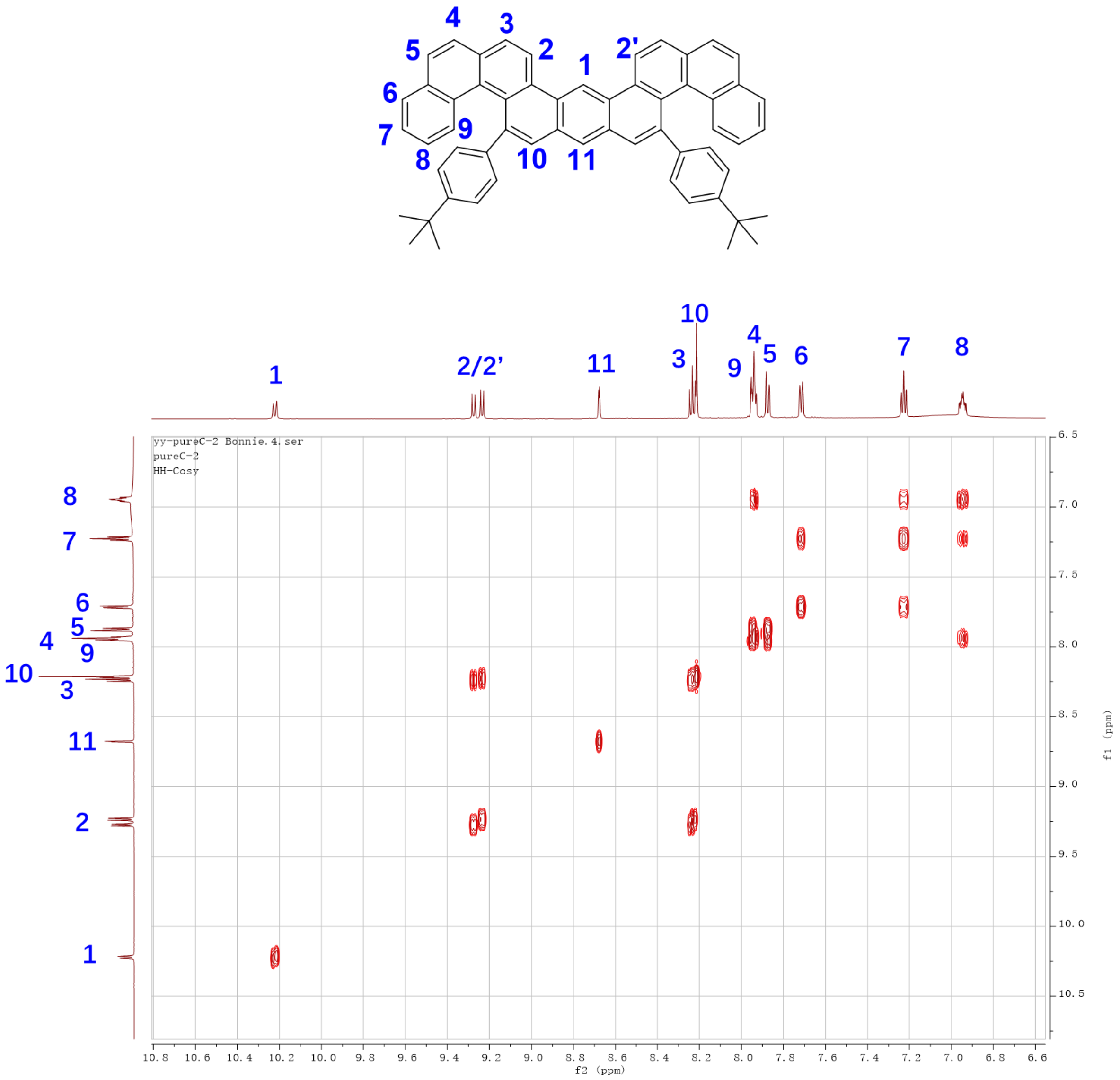

Figure S18. The ^{1}H/^{1}H COSY spectrum of **2** in $C_2D_2Cl_4$ at room temperature.

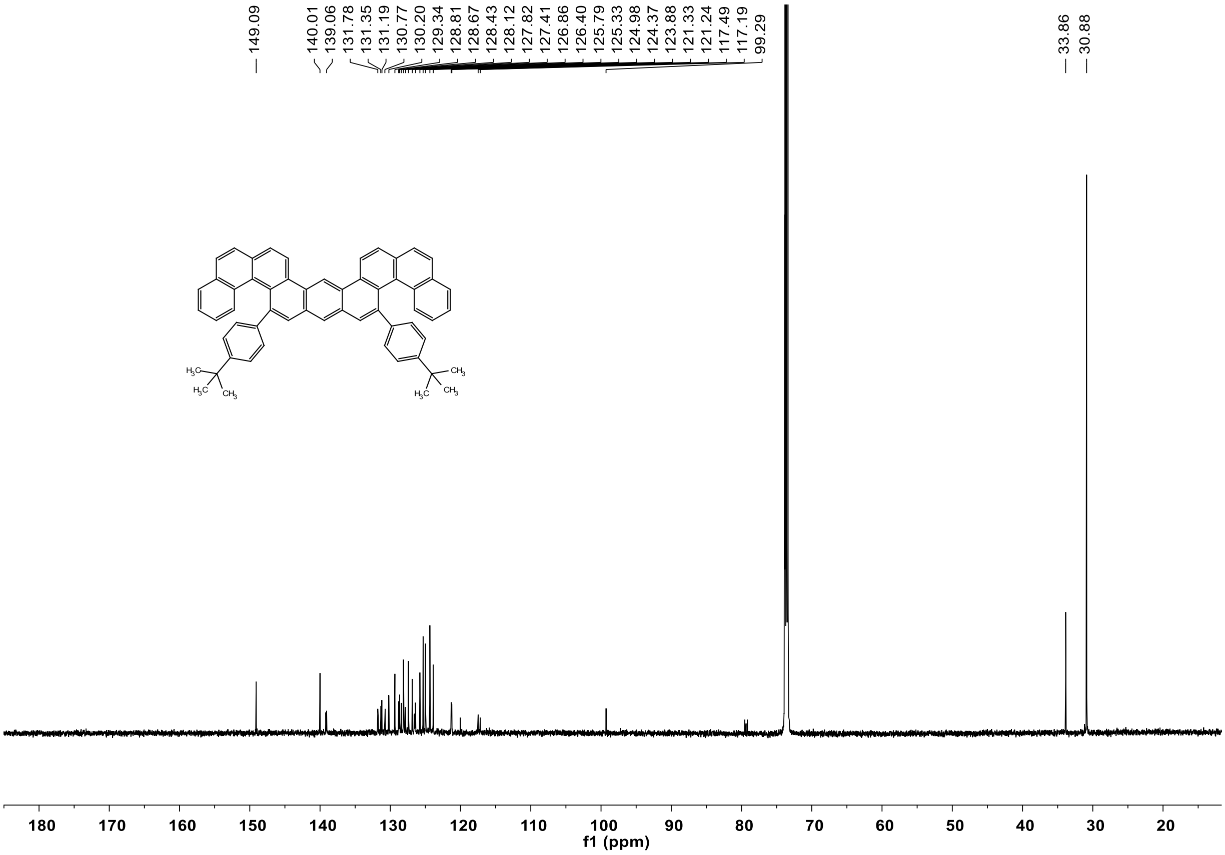

Figure S19. The ^{13}C NMR spectrum (151 MHz) of **2** in $C_2D_2Cl_4$ at room temperature.

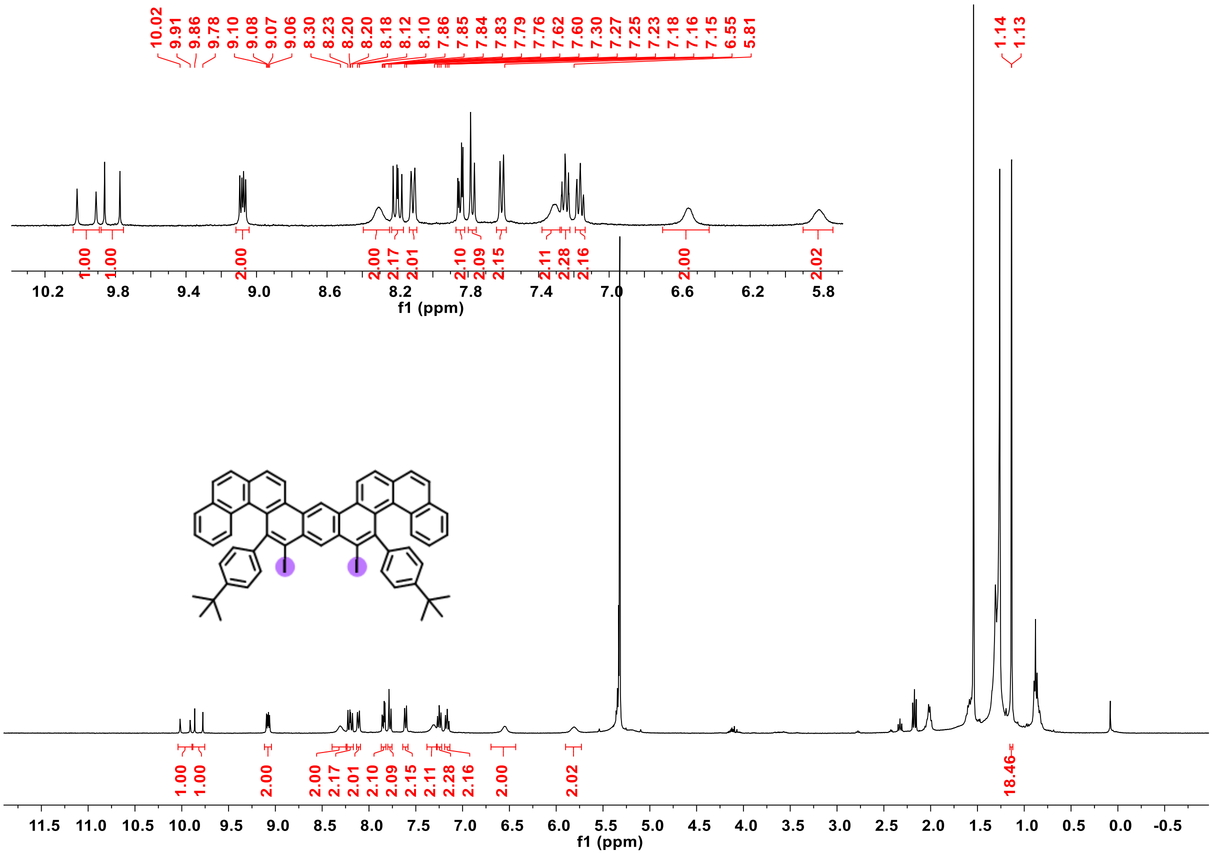

Figure S20. The ^{1}H NMR spectrum (400 MHz) of **3** in CD_2Cl_2 at room temperature.

10. High resolution mass spectrometry

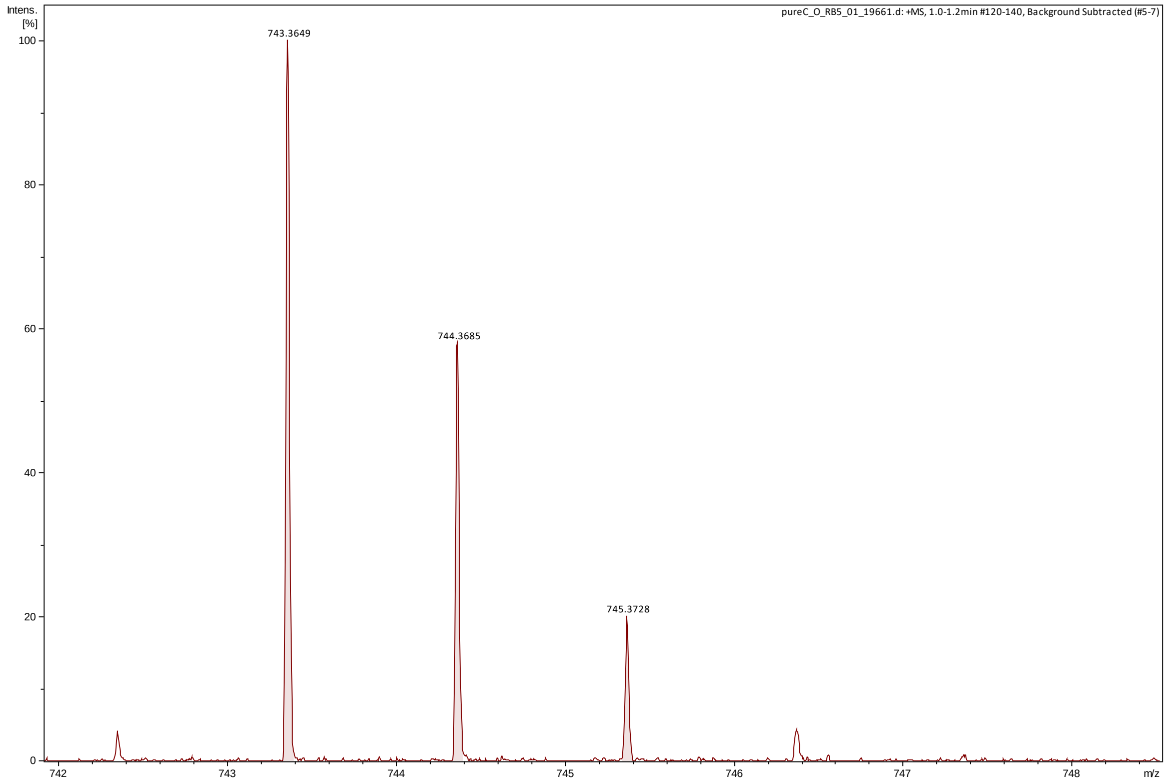

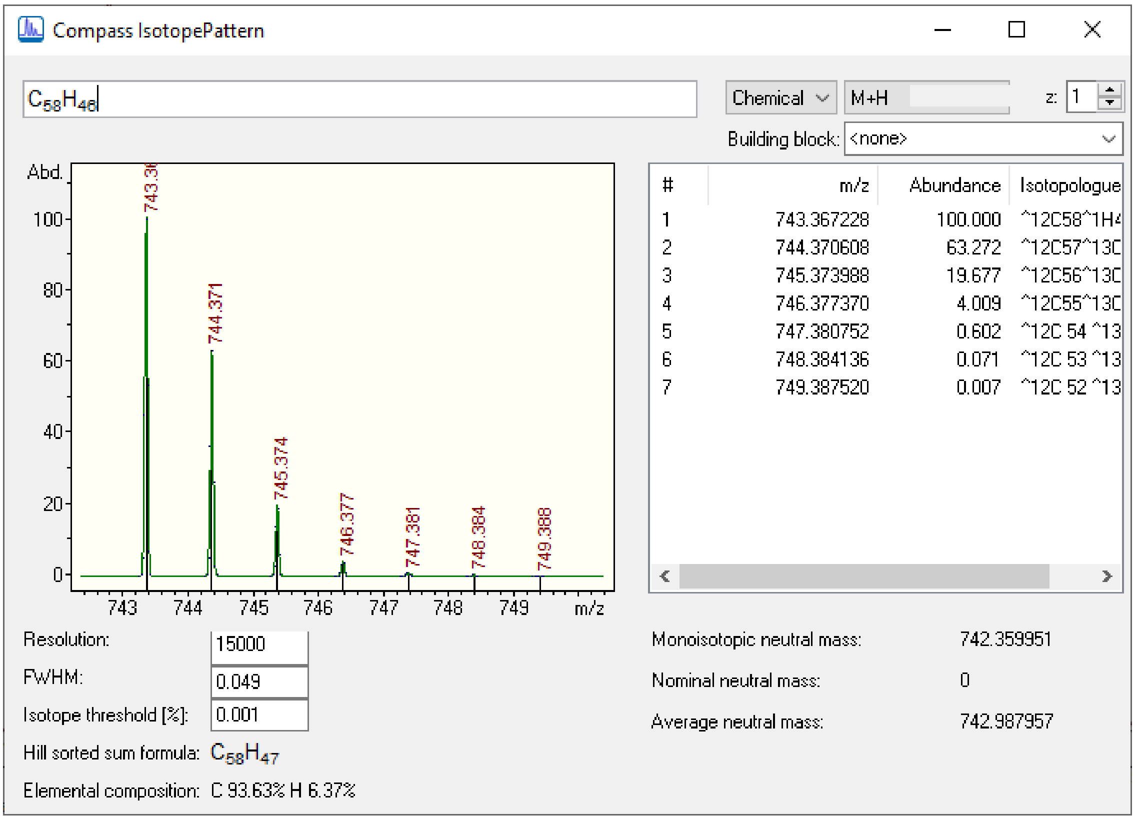

Figure S21. High resolution mass ESI spectrum of **4**.

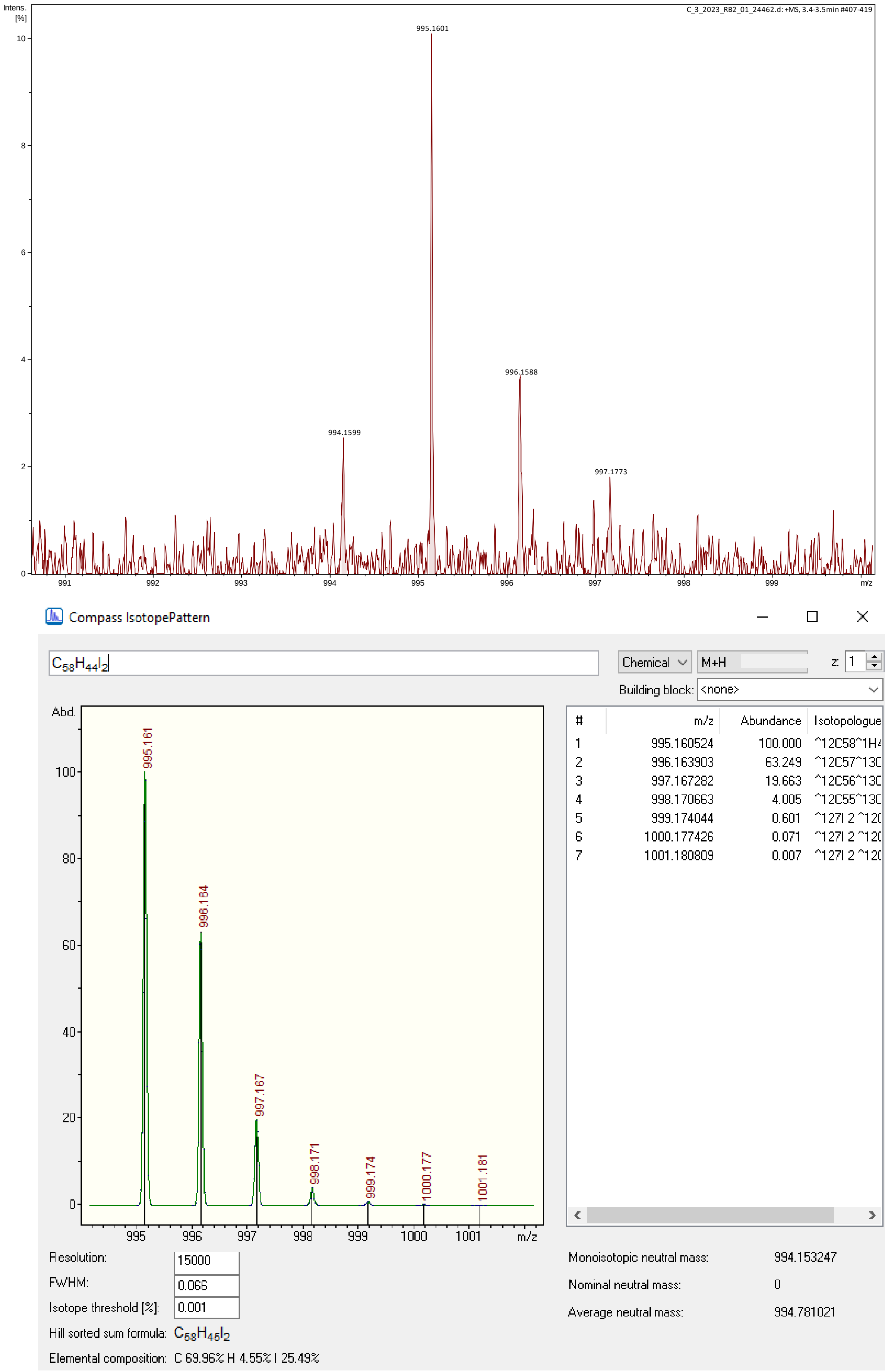

Figure S22. High resolution ESI mass spectrum of **3**.

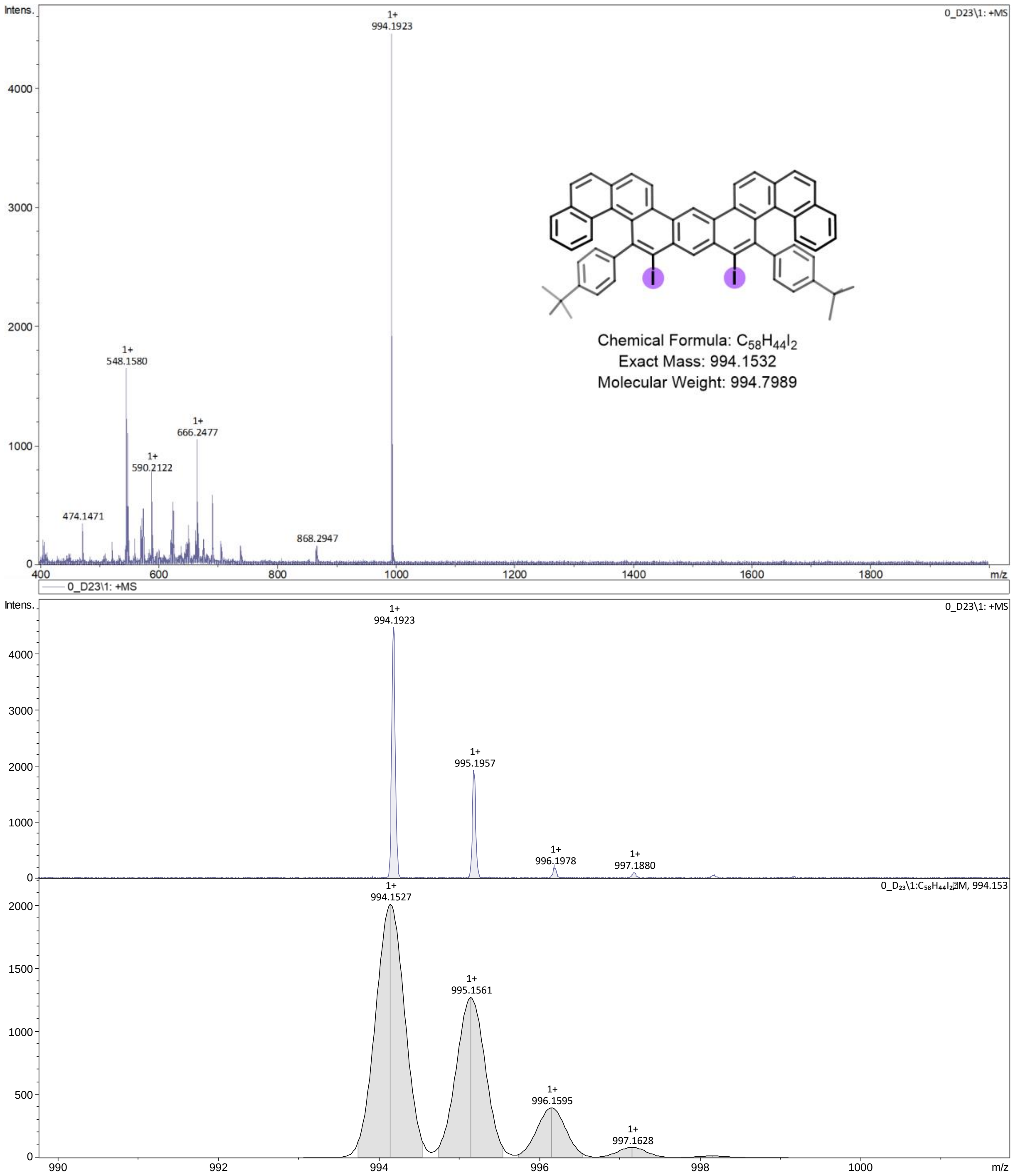

Figure S23. High resolution Maldi-Tof-MS spectrum of **3**.

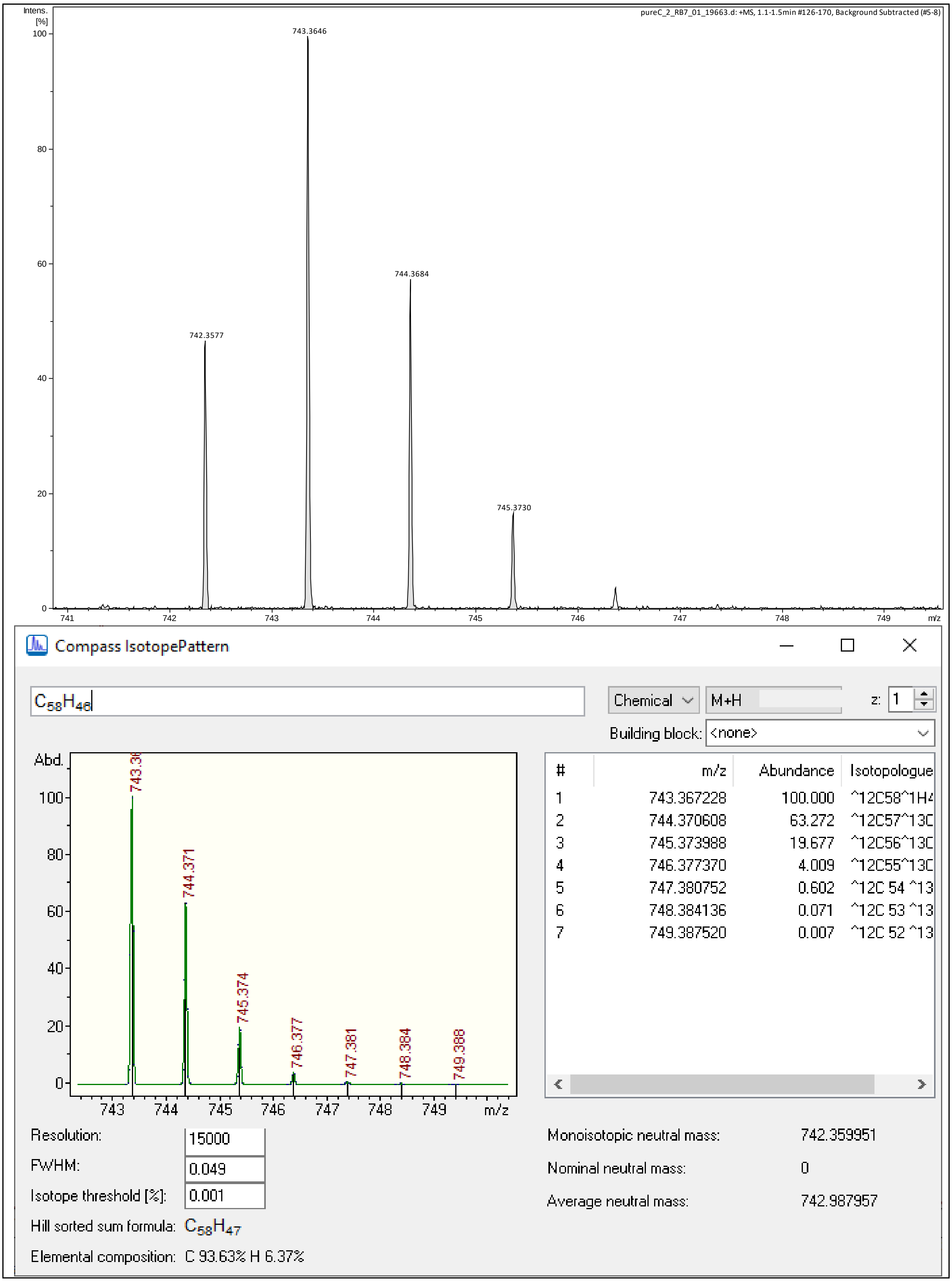

Figure S24. High resolution ESI mass spectrum of **2**.

Full spectrum of **2** shows the monomer and dimer.

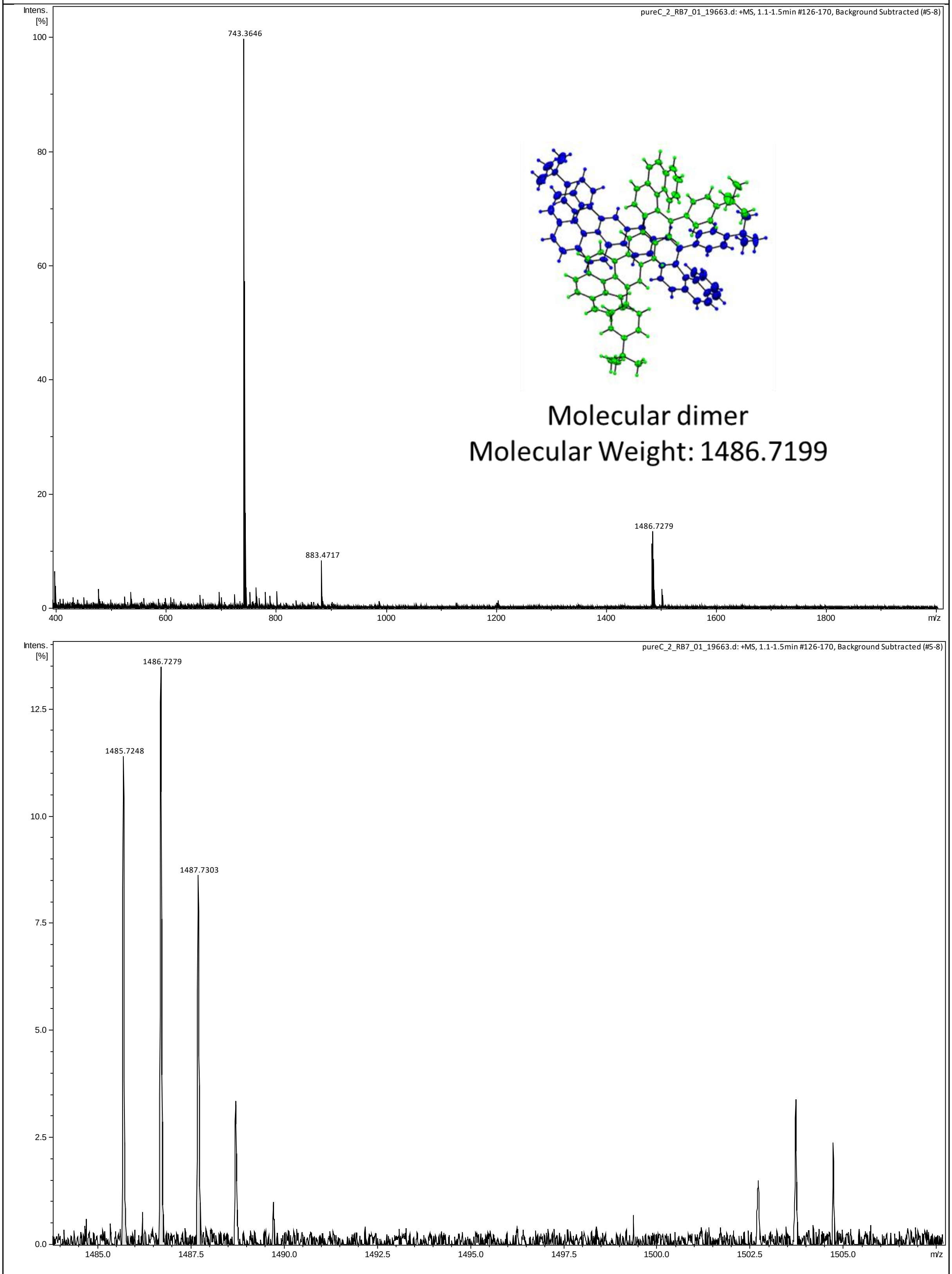

Figure S25. High resolution ESI mass spectrum of the dimer of **2**.

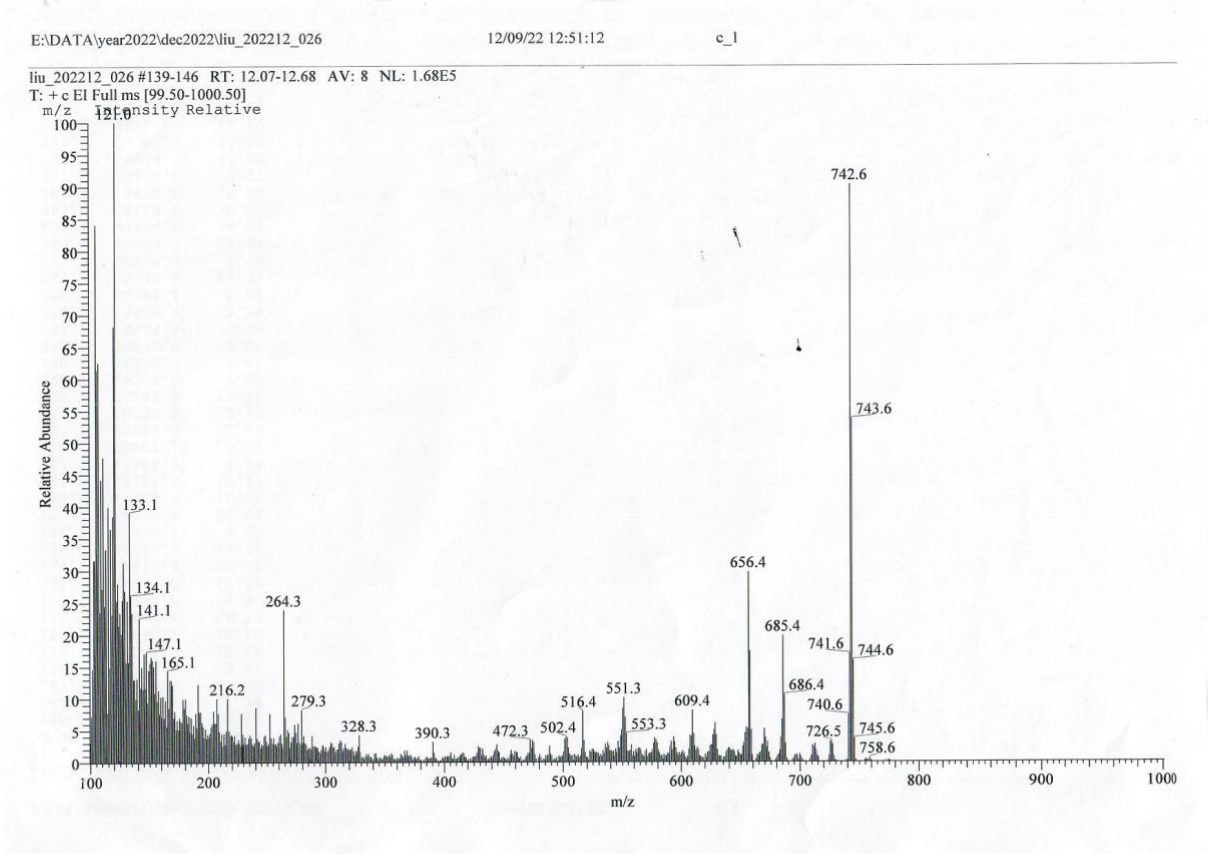

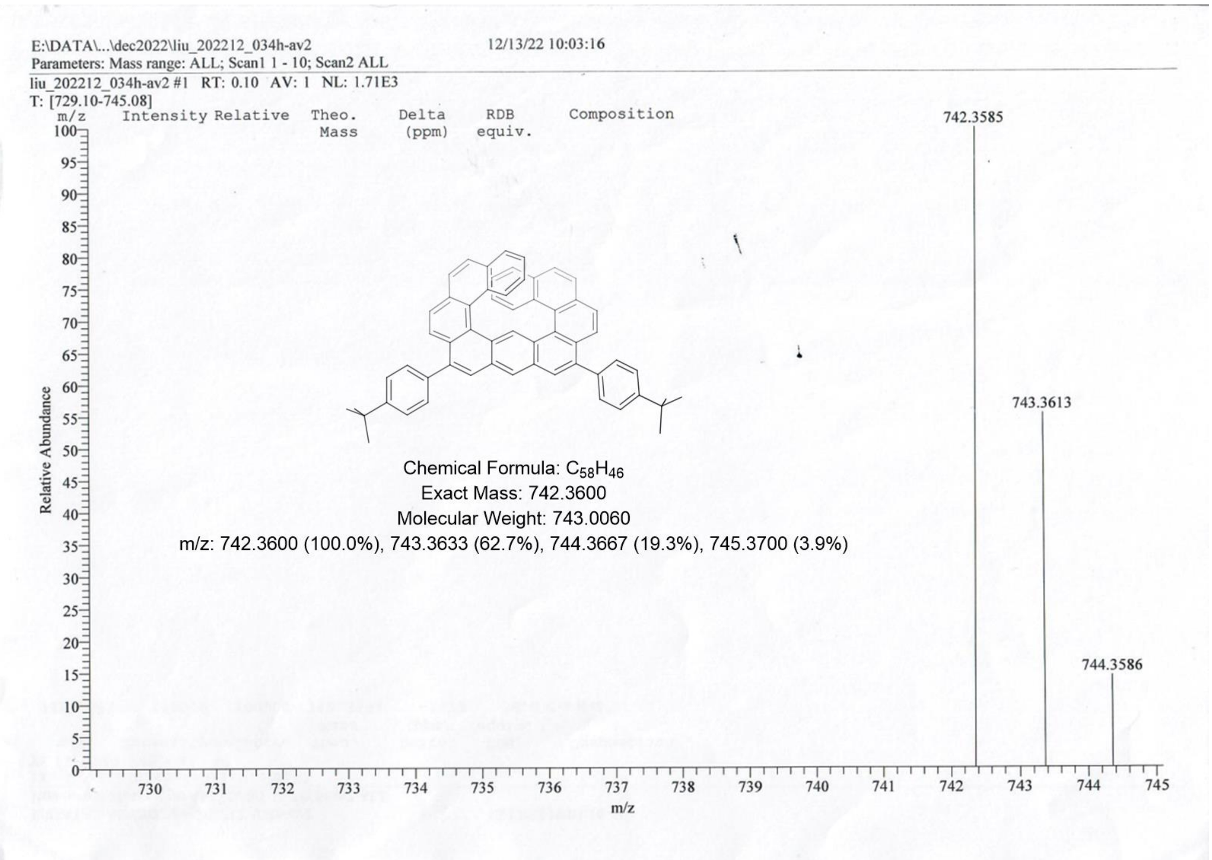

Figure S26. High resolution EI-MS spectrum of **1**.

11. References

[1] J. J. Zhang, M. C. Tang, Y. Fu, K. H. Low, J. Ma, L. Yang, J. J. Weigand, J. Liu, V. W. Yam, X. Feng, *Angew. Chem. Int. Ed.* **2021**, *60*, 2833-2838.

[2] Z. Qiu, C. W. Ju, L. Frederic, Y. Hu, D. Schollmeyer, G. Pieters, K. Mullen, A. Narita, *J. Am. Chem. Soc.* **2021**, *143*, 4661-4667.

[3] D. Geuenich, K. Hess, F. Köhler, R. Herges, *Chem. Rev.* **2005**, *105*, 3758-3772.

[4] K. Wolinski, J. F. Hinton, P. Pulay, *J. Am. Chem. Soc.* **1990**, *112*, 8251-8260.

[5] P. v. R. Schleyer, C. Maerker, A. Dransfeld, H. Jiao, N. J. R. van Eikema Hommes, *J. Am. Chem. Soc.* **1996**, *118*, 6317-6318.

[6] Z. Chen, C. S. Wannere, C. Corminboeuf, R. Puchta, P. v. R. Schleyer, *Chem. Rev.* **2005**, *105*, 3842-3888.

[7] L. Zhang, T. Wu, Y. Guo, Y. Zhao, X. Sun, Y. Wen, G. Yu, Y. Liu, *Sci. Rep.* **2013**, *3*, 1080.

[8] S. A. Lee, D. Y. Kim, K. U. Jeong, S. H. Lee, S. Bae, D. S. Lee, G. Wang, T. W. Kim, *Org. Electron.* **2015**, *27*, 18-23.

[9] W. Y. Lee, H. C. Wu, C. Lu, B. D. Naab, W. C. Chen, Z. Bao, *Adv. Mater.* **2017**, *29*. 1605166.

[10] C. Sun, Z. Q. Lin, W. J. Xu, L. H. Xie, H. F. Ling, M. Y. Chen, J. Wang, Y. Wei, M. D. Yi, W. Huang, *J. Phys. Chem. C* **2015**, *119*, 18014-18021.

[11] Y. Yu, L. Y. Bian, J. G. Chen, Q. H. Ma, Y. X. Li, H. F. Ling, Q. Y. Feng, L. H. Xie, M. D. Yi, W. Huang, *Adv. Sci.* **2018**, *5*, 1800747.

[12] C. M. Tran, H. Sakai, Y. Kawashima, K. Ohkubo, S. Fukuzumi, H. Murata, *Org. Electron.* **2017**, *45*, 234-239.

[13] J. Aimi, C. T. Lo, H. C. Wu, C. F. Huang, T. Nakanishi, M. Takeuchi, W. C. Chen, *Adv. Electron. Mater.* **2016**, *2*, 1500300.

[14] J. Aimi, P.-H. Wang, C.-C. Shih, C.-F. Huang, T. Nakanishi, M. Takeuchi, H.-Y. Hsueh, W.-C. Chen, *J. Mater. Chem. C* **2018**, *6*, 2724-2732.

[15] H. C. Chang, C. Lu, C. L. Liu, W. C. Chen, *Adv. Mater.* **2015**, *27*, 27-33.

[16] W. Li, F. Guo, H. Ling, H. Liu, M. Yi, P. Zhang, W. Wang, L. Xie, W. Huang, *Small* **2018**, *14*, 1701437.

[17] W. Li, F. Guo, H. Ling, P. Zhang, M. Yi, L. Wang, D. Wu, L. Xie, W. Huang, *Adv. Sci.* **2017**, *4*, 1700007.

[18] C. Zheng, T. Tong, Y. Hu, Y. Gu, H. Wu, D. Wu, H. Meng, M. Yi, J. Ma, D. Gao, W. Huang, *Small* **2018**, *14*, 1800756.

[19] B. Y. Jiang, S. Vegiraju, A. S. T. Chiang, M. C. Chen, C. L. Liu, *J. Mater. Chem. C* **2017**, *5*, 9838-9842.

[20] K. Pei, X. Ren, Z. Zhou, Z. Zhang, X. Ji, P. K. L. Chan, *Adv. Mater.* **2018**, *30*, 1706647.

[21] Y.-C. Chiu, T.-Y. Chen, Y. Chen, T. Satoh, T. Kakuchi, W.-C. Chen, *ACS Appl. Mater. Interfaces.* **2014**, *6*, 12780-12788.

[22] Y. Park, K. J. Baeg, C. Kim, *ACS Appl. Mater. Interfaces* **2019**, *11*, 8327-8336.

[23] Y. C. Chiang, C. C. Hung, Y. C. Lin, Y. C. Chiu, T. Isono, T. Satoh, W. C. Chen, *Adv. Mater.* **2020**, *32*, e2002638.

[24] Q. Li, T. Li, Y. Zhang, Z. Chen, Y. Li, L. Jin, H. Zhao, J. Li, J. Yao, *J. Phys. Chem. C* **2020**, *124*, 23343-23351.

[25] M. Higashinakaya, T. Nagase, H. Abe, R. Hattori, S. Tazuhara, T. Kobayashi, H. Naito, *Appl. Phys. Lett.* **2021**, *118*, 103301.

[26] D.-W. Liu, Y. Zhang, X.-Y. Li, Q. Xiao, W.-J. Sun, X. Shao, H.-L. Zhang, *J. Mater. Chem. C* **2021**, *9*, 6560-6567.

[27] W. V. Wang, Y. Zhang, X. Y. Li, Z. Z. Chen, Z. H. Wu, L. Zhang, Z. W. Lin, H. L. Zhang, *InfoMat* **2021**, *3*, 814-822.

[28] J. Zhang, M. Xie, Y. Xin, C. Han, L. Xie, M. Yi, H. Xu, *Angew. Chem. Int. Ed.* **2021**, *60*, 24894-24900.

[29] T. Xu, S. Fan, M. Cao, T. Liu, J. Su, *Appl. Phys. Lett.* **2022**, *120*, 073301.

[30] Y. Yang, Z. Li, C. Wu, W. Li, J. Wang, M. Yi, W. Huang, *J. Mater. Chem. C* **2022**, *10*, 3292-3299.

[31] Y. J. Jeong, D. J. Yun, S. H. Noh, C. E. Park, J. Jang, *ACS nano* **2018**, *12*, 7701-7709.

[32] K. Thakur, D. Wang, S. V. Lindeman, R. Rathore, *Chem. Eur. J.* **2018**, *24*, 13106-13109.

[33] R. Kusy, K. Grela, *Green Chemistry* **2021**, *23*, 5494-5502.
